# Design and Construction Practices for Full-Depth Reclamation of Asphalt Mixtures with Bituminous and Cementitious Additives

**DOI:** 10.3390/ma19081540

**Published:** 2026-04-12

**Authors:** Swathi Malluru, Ahmed Saidi, Ayman Ali, Yusuf Mehta

**Affiliations:** Center for Research and Education in Advanced Transportation Engineering Systems (CREATES), Rowan University, Glassboro, NJ 08028, USA; ahmeds@rowan.edu (A.S.); alia@rowan.edu (A.A.); mehta@rowan.edu (Y.M.)

**Keywords:** full-depth reclamation, rehabilitation, cement, asphalt emulsion, foamed asphalt, mix design

## Abstract

**Highlights:**

- Synthesized current full-depth reclamation (FDR) mix design and construction practices adopted by highway agencies through literature review, survey and subject matter expert.
- Evaluated the laboratory performance of FDR mixtures with Portland cement, asphalt emulsion, and foamed asphalt in terms of rutting and cracking susceptibility using Asphalt Pavement Analyzer (APA) and indirect tensile (IDT) tests, respectively.
- The APA and IDT tests can be used effectively to select the optimum dosages of bituminous and cementitious additives.
- FDR mixtures stabilized with asphalt emulsion improved rutting and cracking performance compared to cement-only and foamed asphalt mixtures.

**Abstract:**

Several highway agencies have implemented full-depth reclamation (FDR) as a sustainable technology for rehabilitating deteriorated asphalt pavements. However, the lack of standardized mix design procedures and limited field assessment, in terms of rutting and cracking resistance, pose challenges to the widespread implementation of FDR. This study addresses these challenges by synthesizing current FDR mix design and construction practices and validating highway agency-recommended practices through laboratory performance evaluation. The study objectives were achieved by (1) reviewing current FDR mix design and construction specifications of highway agencies across the US and internationally, (2) conducting surveys with highway agencies and interviews with subject matter experts (SMEs), and (3) evaluating the laboratory performance of FDR mixtures. Based on the findings from the literature, survey responses, and SME interviews, three FDR mixtures were designed in the lab: (i) cement-only, (ii) asphalt emulsion and cement, and (iii) foamed asphalt and cement. Each mix was then evaluated for rutting susceptibility using the Asphalt Pavement Analyzer (APA) and cracking resistance using the indirect tensile (IDT) test to identify optimum dosages of bituminous and cementitious additives. Laboratory results showed that FDR mixtures with 3% asphalt emulsion and 1% cement improved rutting resistance by 46% and cracking performance by 70% compared to cement-only mixtures with 4% cement. In contrast, foamed asphalt did not result in a significant improvement in FDR performance. Survey responses indicated that 89% of respondents reported good field performance of FDR, with Pennsylvania and North Dakota exhibiting excellent performance 10 years after construction.

## 1. Introduction

The need for capacity improvements, environmental regulations, budget constraints, scarcity of materials, and rising material and transportation costs has significantly increased the demand for cost-effective, sustainable, and durable pavement rehabilitation techniques [1]. To address these challenges, highway agencies are recycling damaged asphalt pavements using in-place recycling techniques as a rehabilitation strategy [2]. FDR rehabilitation technology provides a more comprehensive approach for recycling severely deteriorated asphalt pavements and proven to be cost-effective and technically feasible. This process effectively addresses deep and severe structural deficiencies, including base failures, by reclaiming the asphalt pavement with a pulverization depth of up to 14 in [3]. As such, FDR materials typically include the asphalt surface layer, the underlying base, and unbound subgrade soils. The stabilized FDR base layer is produced by uniformly pulverizing the existing asphalt pavement and blending with a predetermined portion of underlying materials, and stabilizing agents (often referred to as additives) [4,5]. Chemical additives such as Portland cement, hydrated lime, calcium chloride, and fly ash, and bitumen additives including asphalt emulsion and foamed asphalt are added to the mixture to provide adequate structural strength [6,7,8].

Previous studies reported that FDR is a more cost-effective rehabilitation solution than other rehabilitation technologies, as it reduces the need for virgin aggregates and minimizes the disposal of existing pavement materials. For instance, a study by Torres-Machi et al. evaluated the cost-effectiveness and long-term performance of FDR and overlay pavements and found that FDR-rehabilitated pavement reduced the life cycle cost by $178,243 per lane-mile, representing a potential 30% savings when compared to the conventional overlay solution [9]. Braham [10] found that cement- and bitumen-stabilized FDR pavements saved 51% to 71% compared to traditional reconstruction. In addition to economic benefits, the study also reported a 32.2% increase in the long-term performance of FDR pavements compared to overlay [9]. Haiwei Zhang et al. highlighted the environmental benefits of FDR and demonstrated that carbon emissions from FDR with Portland cement were 92% and 90% relative to the conventional remove and reconstruction, and cold central plant recycling (CCPR) technology, respectively, while the energy consumption levels were merely at 40% and 60% of the latter two technologies [11].

Several highway agencies, including Virginia, Pennsylvania, California, Georgia, Nevada, Texas, Maine, Idaho, Minnesota, South Carolina, and technical organizations such as the Asphalt Recycling and Reclaiming Association (ARRA) and the Asphalt Academy developed guidelines for laboratory mix design, construction practices, and quality control procedures for field implementation. However, considerable variations exist among these mix design procedures, performance evaluation methods, construction practices, and quality control procedures for field implementation [12,13,14]. Current mix design methods differ in compaction techniques, curing processes, moisture and stabilizer contents, performance measures, and criteria for selecting stabilizer dosages.

The inconsistencies in FDR design and construction practices across the highway agencies can be attributed to several factors including climate and geographical location, difference in RAP and soil properties, contractor experience, and equipment availability. Climate and geographic location play a significant role in determining the performance criteria. For instance, states in colder regions such as Minnesota and North Dakota prioritize flexibility and cracking resistance to withstand freeze–thaw cycles, while southern states including Texas and Georgia focus on rutting resistance at high temperatures. The composition of RAP and soil materials varies considerably across the regions, meaning the mix design developed in one state may not perform the same when used elsewhere. Highway agencies with limited contractor experience and equipment unavailability tend to adopt simpler approaches to reduce the construction risk, while experienced agencies develop standard specifications with quality control procedures. These inconsistencies and the lack of standardized procedures pose significant challenges for agencies in selecting mix design and construction practices for FDR implementation, thereby limiting its broader adoption as a rehabilitation technology [15,16,17].

In addition, inadequate structural design methods, limited quality control practices, and insufficient long-term field performance data further act as a potential barrier for agencies employing FDR [9,15,16]. While prior studies, such as National Cooperative Highway Research Program (NCHRP) Synthesis [16], provided comprehensive overviews of current FDR practices based primarily on survey responses, there is a need to validate these practices with the laboratory performance for broader implementation. The present study addresses this need by synthesizing current agency specifications, survey responses, and SME interviews, and by evaluating the laboratory performance of cement- and bitumen-stabilized FDR mixtures. This integrated approach can provide agencies with a more consistent mix design methodology for broader implementation of FDR, while allowing project-specific materials and conditions.

## 2. Goal and Objectives

The primary goal of this study is to evaluate the laboratory performance of FDR mixtures with cementitious and bituminous additives and to determine the optimum dosages based on rutting and cracking performance. In addition, the study synthesizes current FDR mix design and construction practices to inform the experimental program. To achieve these goals, the following objectives were identified:

- Review current FDR mix design and construction specifications adopted by highway agencies in the US and internationally.
- Collect and analyze responses from survey questionnaires and SME interviews from highway agencies across the US experienced in FDR implementation.
- Identify the widely used stabilizing agents, dosage ranges, compaction methods, curing conditions, performance tests, and evaluation criteria for FDR mixtures based on literature, survey responses, and SME recommendations.
- Design representative FDR mixtures with bituminous and cementitious additives based on the synthesized findings.
- Evaluate the rutting and cracking susceptibility of the FDR mixtures over a range of dosages using APA and IDT, respectively.
- Determine the optimum dosage of stabilizers based on a balanced rutting and cracking performance.

## 3. Methodology

**Figure 1:**
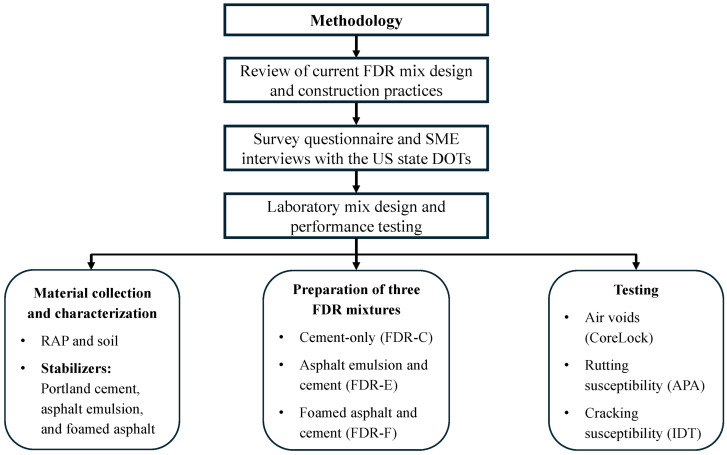
Research approach of the study.

The study was conducted in three steps to evaluate the laboratory performance of FDR with bituminous and cementitious additives based on rutting and cracking resistance. Figure 1 presents the methodology adopted for the study. As can be seen from Figure 1, technical reports, peer-reviewed journal articles, and agency specifications on FDR were collected from various US state departments of transportation (DOTs) and databases including TRB, Scopus, and ASCE library, published between 1985 and 2025, to capture agencies’ experience with FDR implementation, and a 41-question survey was distributed through Qualtrics XM Platform to all the state DOTs, focusing on current practices, stabilizing agents’ selection, performance evaluation methods, and mix design and construction practices.

In addition, SMEs from academic institutions and highway agencies were interviewed via Zoom 6.0 to gain insights on FDR challenges in field implementation and lessons learned. The compiled agency practices, survey and interview responses were synthesized to define representative mixture design and experimental conditions for laboratory testing of FDR mixtures stabilized with bituminous and cementitious stabilizers. Based on the synthesis, representative stabilizer type, dosage ranges, water content, compaction method, curing conditions, performance tests, and performance criteria were identified. Three FDR mixtures: (1) cement-only (FDR-C), (2) asphalt emulsion and cement (FDR-E), and (3) foamed asphalt and cement (FDR-F) were selected for laboratory performance evaluation in terms of rutting and cracking resistance. The optimum dosage of stabilizer for each mixture was determined as the combination that minimized the rut depth and maximized the ITS. In this study, the cement-only mixture was considered as the control mix for comparing the performance of cement-stabilized and bitumen-stabilized mixtures. Overall, this approach developed a comprehensive framework for FDR best practices by integrating literature, practical experience, and laboratory evaluation.

## 4. Synthesis of FDR Mix Design and Construction Practices

This study compiles the current FDR design specifications adopted by various highway agencies in the US. This section focuses on mix design procedures, material characterization, selection of stabilizing agents and dosages, water content, compaction methods, curing process, laboratory performance evaluation criteria, and construction practices. The findings form the basis for selecting the materials and mix design parameters for the laboratory experimental program.

### 4.1. Laboratory Mix Design Procedures

Several highway agencies (e.g., VDOT, PennDOT, IDOT, TDOT, CDOT, SCDOT, NJDOT, NYDOT, and NDOT) and organizations (e.g., Asphalt Recycling and Reclaiming Association (ARRA), Wirtgen, Asphalt Academy, and American Association of State Highway and Transportation Officials (AASHTO)) developed individual mix design procedures for FDR with many similarities and certain differences in reclaimed asphalt pavement (RAP) gradation, binder type, compaction, curing, and design criteria. ARRA and AASHTO [17,18,19,20,21] developed mix design procedures for cement and asphalt emulsion-stabilized FDR mixtures, while Wirtgen and Asphalt Academy developed for cold recycled mixtures.

**Figure 2:**
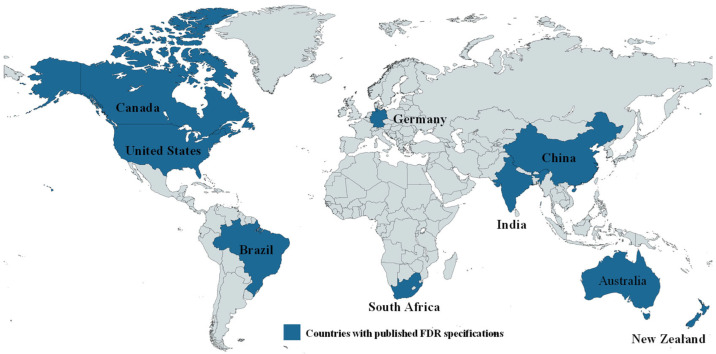
Countries with documented specifications for FDR.

**Figure 3:**
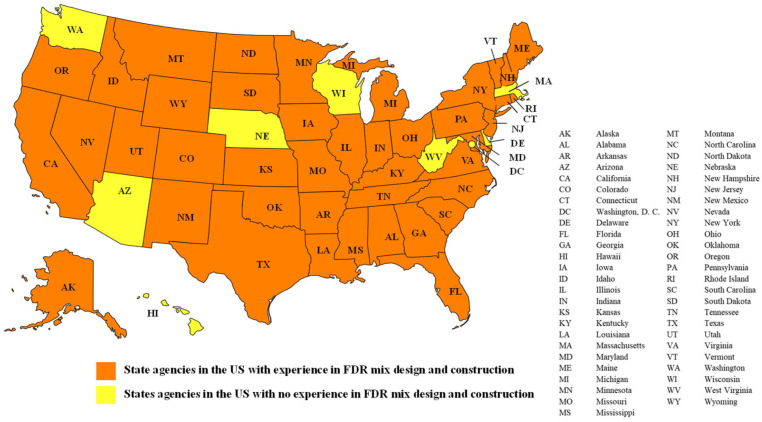
Geographical representation of US states with and without experience in FDR.

Figure 2 presents the map of countries with published specifications for FDR. As can be seen from Figure 2, several countries across the continents, including the United States, Canada, Brazil, South Africa, Germany, India, China, Australia, and New Zealand, have developed specifications for FDR implementation. Figure 3 illustrates the US states with experience in FDR implementation. From Figure 3, the 41 states highlighted in orange have experience with FDR implementation, while the 9 states in yellow have no active field implementation. These figures indicate the current adoption of FDR as a rehabilitation technique both nationally and globally, with many highway agencies currently implementing FDR and developing specifications for its adoption.

The data presented in Figure 2 was compiled by the authors through a comprehensive review of published literature, official agency websites and documents, and publicly available resources. In addition, Figure 3 was developed by compiling a comprehensive review of official State DOT websites and direct communication with the agencies to verify the availability of FDR specifications and practices.

#### 4.1.1. Material Sampling and Characterization

RAP and soil materials for FDR mix design are obtained by reclaiming or coring a portion from the HMA, base, and subgrade layers. When collecting cores, samples are cored to the target depth corresponding to the planned FDR pulverization depth, representing the milling process (i.e., up to 14 in.) using a coring rig. Sieve analysis is conducted on RAP and soil materials to determine the maximum particle size and fines contents, as the maximum particle size significantly affects mix compactability, while the fines percentage influences stiffness. Both Pennsylvania and Virginia DOTs recommend 95% and 100% passing the 2 in. sieve, respectively, for FDR mixtures [22,23]. Maine and Colorado DOTs require 100% RAP passing a 2-inch square mesh sieve [24,25]. ARRA specifies RAP to be crushed to 100% passing the 1.5 in. sieve [17,18,19,20]. The Portland cement association (PCA) and ARRA recommend a minimum of 20% passing the No. 200 sieve to minimize the quantity of stabilizing agents and rehabilitation cost [26].

#### 4.1.2. Selection of Stabilizing Agents

To improve structural properties, durability, and long-term performance of FDR-rehabilitated pavements, the layers are generally treated with one of the three stabilization methods: (1) chemical stabilization using Portland cement, lime slurry, fly ash or calcium chloride, (2) bituminous stabilization using asphalt emulsion or foamed asphalt, and (3) mechanical stabilization through the addition of virgin aggregates [26]. The choice of a stabilizing agent and its dosage significantly affects the strength and performance of FDR mixtures and depends on many factors, including RAP and soil characteristics, thickness of existing pavement, and contractors’ experience [12]. Inadequate stabilizing agent can result in poor aggregate binding within the mixture, while excessive dosage can increase rigidity and lead to cracking [27].

Portland cement or lime are commonly used chemical additives in FDR mixtures to increase strength, cohesion, moisture damage resistance, and decrease curing time. Portland cement with low heat of hydration is recommended to avoid shrinkage [28]. Although cement content increases the strength of FDR mixtures, higher cement content may also increase susceptibility to shrinkage cracking [29]. In general, three cement contents are tested for FDR mix design, based on AASHTO soil classification, as presented in the Soil-Cement Laboratory Handbook [30]. For instance, if the soil is A-1-b type (maximum 25% passing No. 200 sieve), 3%, 4%, and 5% cement by weight are tested for compressive strength [31]. California DOT recommends at least 1% Portland cement for acid crystalline pulverized material, while both cement and lime are used for basic crystalline materials [28]. Previous studies [32,33,34] have shown that cement contents within the 4–6% range provide adequate strength development and optimized performance for FDR mixtures, supporting the dosage range selected in this study.

Asphalt emulsion and foamed asphalt are the most commonly used bituminous stabilizers in FDR mixtures as specified by different highway agencies. Asphalt emulsion enhances the cohesion and load-bearing capacity of mixtures while rejuvenating and softening aged RAP binder [35]. A cationic or anionic type of asphalt emulsion is selected depending on RAP compatibility, binding properties, coating, strength, and asphalt emulsion breaking time. CSS-1h (cationic slow setting), HFMS-2 (high-float medium-setting with a solvent), and HFMS-2p (high-float medium-setting modified with a polymer) are commonly used asphalt emulsions, with slow setting being widely used [36,37]. Foamed asphalt is characterized by expansion ratio and half-life, which determines the optimum foaming water content (OFWC) [38]. The minimum foaming characteristics for FDR mixtures are (1) 10 times expansion ratio and 8 s half-life for RAP temperature ranging between 10 °C and 15 °C, and (2) 8 times expansion ratio and 6 s half-life when RAP temperature is above 15 °C [23,39]. Mallick et al. [40] reported that FDR mixes with asphalt emulsion and cement attain strength faster and exhibit high shear strength and moisture damage resistance compared to mixes with only water. Table 1 presents the comparison of stabilizing agents used for FDR mixtures.

#### 4.1.3. Selection of Water Content

Water is an essential component in cold recycled mixtures for coating RAP with stabilizers and facilitating compaction [45]. The optimum water content (OWC) of FDR is obtained from standard or modified Proctor density tests. Agencies including VDOT and PennDOT use Standard Proctor test in accordance with AASHTO T134 [46] to determine the OWC of cement-stabilized mixtures and Modified Proctor test in accordance with AASHTO T180 [47] for bitumen-stabilized mixtures. New York DOT [48] specifies a constant water content of 3% by total mix weight when RAP is above 60%, and 2–3% when the percentage passing No. 200 is less than 4. The selection of water content varies across highway agencies, with some adopting Proctor test, while others use constant water content for bitumen-stabilized FDR mixtures. These variations affect the stabilization mechanism. In cement-stabilized mixtures, water governs cement hydration and strength development, whereas in bitumen-stabilized mixtures, water affects compaction, binder dispersion, and strength gain. The lack of a standardized approach in determining the OWC highlights a significant gap in current FDR mix design practices.

#### 4.1.4. Compaction

The selection of compaction technique depends on the type of bituminous additives, which in turn affects the performance and durability of mixtures. Mallick et al. [49] recommended 50 gyrations to achieve a minimum of 98% of the Modified Proctor density in the field. Kim et al. [50] reported that 30 gyrations are comparable to 75 Marshall blows and suggested adopting the Superpave gyratory compactor (SGC) for FDR with emulsified or foamed asphalt. TxDOT specifies a target compaction height of 2 in. for asphalt emulsion-stabilized mixtures and 3.75 in. for foamed asphalt mixtures [51]. Prior to compacting asphalt emulsion-stabilized FDR mixtures, ARRA and AASHTO recommend allowing them to cure at 40 °C for 30 + 3 min [17,21]. Cement-stabilized FDR mixtures are stored at 25 °C + 4 °C to 30 gyrations before compaction [19]. Once cured, each mix is immediately compacted at 25 °C + 4 °C to 30 gyrations using an SGC [17]. These variations in compaction methods across agencies introduce uncertainty in evaluating mixture performance, highlighting a knowledge gap in standardizing compaction method for FDR mixtures.

#### 4.1.5. Curing

Many researchers tested various curing times, temperatures, and humidity levels to accelerate the development of long-term properties and simulate field curing conditions for cold asphalt mixtures [52,53]. Yahya et al. [54] reported that high temperature and low humidity curing improved mechanical properties like tensile strength and stiffness modulus, whereas high humidity decreased performance. Several studies have used curing at 60 °C for 72 h as a laboratory procedure for curing cold recycled mixtures, to represent medium or long-term field curing [53]. While Marquis et al. [55] recommended a curing process of 40 °C for 72 h for foamed asphalt stabilized FDR mixtures. ARRA and AASHTO specify curing asphalt emulsion-stabilized, compacted specimens at 60 ± 1 °C until a constant mass is achieved, defined as a change in mass of 0.05% or less within 2 h, with a total curing period between 16 and 48 h [17,21]. However, Asphalt Academy [13] recommends curing the bituminous stabilized FDR mixtures at 40 °C ± 1 °C for minimum 72 h after compaction. These variations in curing temperature reflect differences in the stabilization mechanism of bituminous mixtures. A lower curing temperature of 40 °C is typically used for foamed asphalt to simulate field conditions and avoid excessive loss of moisture. In contrast, a higher curing temperature of 60 °C is adopted for asphalt emulsion-stabilized mixtures to accelerate moisture loss and promote early strength gain. These inconsistencies in curing conditions highlight the absence of a unified curing protocol for bituminous-stabilized FDR mixtures and indicate a key knowledge gap in correlating laboratory conditions with field performance.

#### 4.1.6. Selection of Laboratory Tests to Determine Optimum Dosages of Stabilizing Agents

After curing, FDR mixtures with bituminous additives exhibit viscoelastic and performance characteristics similar to those of asphalt concrete. Both asphalt emulsion and foamed asphalt mixtures exhibit similar strength and stiffness properties, leading to the adoption of common performance criteria for these two additives [56].

To characterize the FDR mixtures and to select the optimum dosage of stabilizing agents, numerous researchers conducted laboratory tests, including Marshall stability, indirect tensile (IDT), indirect tensile resilient modulus (IDT Mr), resilient modulus (Mr), triaxial resilient modulus, complex modulus (E*), unconfined compressive strength (UCS), triaxial shear strength, and asphalt pavement analyzer (APA). Marshall stability and IDT tests have been extensively utilized in various studies to evaluate rutting, cracking, and moisture damage susceptibility of FDR mixtures, particularly with bituminous additives [57,58].

Multiple studies widely adopted the IDT test for evaluating the strength of FDR mixtures and monitoring strength development during curing [59,60,61]. Kim et al. [62] conducted both Marshall stability and IDT for the mix design of foamed asphalt mixtures and recommended adopting IDT for determining the optimum dosage of foamed asphalt. A study by Kim et al. [63] employed the IDT test to find the optimum curing time for mixtures with asphalt emulsion and foamed asphalt. The use of IDT and UCS tests in mix design procedures mainly emphasize strength-based performance, while providing limited consideration for rutting susceptibility which is a major distress mechanism in asphalt mixtures [16,64,65]. Cox et al. [66] recommended the APA test for assessing the rutting susceptibility and Cross et al. [67] evaluated the rutting resistance and moisture damage of cold recycled mixtures using APA.

Table 2 highlights the main differences between the FDR specifications of various highway agencies in the US. As can be seen from Table 2, various DOTs specify indirect tensile strength (ITS) as a mix design parameter to assess the strength of FDR mixtures with bituminous additives and to determine the optimum dosage of asphalt emulsion and foamed asphalt, while UCS is specified for cement-stabilized FDR mixtures. Colorado and Maine state DOTs evaluated five criteria: (1) short-term strength test, (2) ITS, (3) conditioned ITS, (4) resilient modulus, and (5) thermal cracking (IDT) for selecting optimum asphalt emulsion content of FDR mixes [68]. After selecting cement and water contents, asphalt emulsion or foamed asphalt is added at varying dosages to pulverized material at OWC and analyzed for air voids, stability, flow, and tensile strength ratio (TSR) for optimum binder content (OBC) [25].

As can be seen from Table 2, most state DOTs recommend RAP with sizes between 1.5 in. and 2 in. Asphalt emulsion (3–6%) and foamed asphalt (2–3%) are used as bituminous additives for FDR mixtures, and Portland cement is used as chemical additive. Most highway agencies specify determining the moisture content from a moisture density curve; however, PennDOT and VDOT recommend a constant water content of 3% and 2%, respectively. INDOT and TxDOT recommend 30 gyrations, whereas VDOT, FDOT, and IDOT suggest either 30 gyrations, or 75 Marshall blows, for compaction. California recommends the Hveem method of compaction for foamed asphalt. Most DOTs specified curing asphalt emulsion or foamed asphalt mixtures at 40 °C for 72 h, while VDOT and MDOT recommend curing at 60 °C to constant mass for FDR mixtures with asphalt emulsion.

The performance tests and strength-based criteria adopted for determining the optimum dosages of stabilizers varies across agencies, with most specifications relying on strength tests including UCS, IDT, and Marshall stability. However, these tests do not capture the key distress mechanisms such as rutting and fatigue cracking. In addition, there is limited or no consideration of rutting susceptibility of FDR mixtures, despite being a primary mode of distress in asphalt mixtures. Furthermore, the choice between ITS and Marshall stability for bituminous stabilized mixtures remains unresolved. These limitations highlight a critical gap in current FDR mix design practices. The present study addresses these gaps by considering both APA and IDT tests for evaluating the FDR mixture performance.

In summary, FDR mix design and construction practices differ from one agency to another and are dependent on the type of stabilizing agent. Specifications vary in the material gradation, materials’ stabilizers’ dosages, compaction, curing, and performance testing. Asphalt emulsion (e.g., CSS-1h) or foamed asphalt (e.g., PG 64-22) are commonly used bituminous additives, and lime or Portland cement as chemical additives. In general, SGC was recommended by various studies and DOTs for compaction, and the ITS test was used for cracking characterization and selecting OBC with minimal emphasis on rutting performance of FDR mixtures. Although numerous studies and specifications are available for FDR, the lack of consistent mix design and construction practices necessitates additional data on construction challenges and practical experiences. As this data is undocumented, there is a need to reach out to highway agencies and SMEs experienced in FDR to gather best practices and lessons learned.

## 5. Outcomes of Survey Questionnaire and SME Interviews

In addition to published agency practices and literature, a 41-question survey was distributed to all US DOTs to collect information on FDR’s current mix design and construction practices. The survey was categorized into three sections: (1) use of FDR to evaluate knowledge, benefits and field implementation; (2) mix design procedures to gather information on RAP properties and collection methods, selection and characterization of bituminous and chemical additives, moisture content, compaction methods, curing periods and performance tests; and (3) construction practices to capture target density, maximum moisture content, type and thickness of overlay, pavement performance, challenges and recommendations. The survey captures practical insights on stabilizer type, dosages, mix design, performance evaluation methods, and construction practices. The outcomes of the survey and SME interviews complement the literature review and help refine the material selection and mix design parameters in the experimental program.

Out of the 50 states, 10 DOTs (Virginia, Pennsylvania, Indiana, Florida, Georgia, New York, Nevada, North Dakota, Arkansas, and Maine) responded to the survey. Although the response rate (20%) was relatively low, the survey covered inputs from diversified climatic and geographic regions across the US. Additionally, the response rate aligns with those reported in similar synthesis studies and NCHRP reports [83,84]. To minimize the non-response bias and enhance dataset representativeness, follow-up emails and phone calls were conducted with non-responding highway agencies. In addition to the survey, SMEs from VDOT, PennDOT, and MNDOT were interviewed to identify FDR’s best design and construction practices, field challenges, recommendations, and lessons learned.

The survey responses highlight variability in various key aspects of FDR design and construction practices, including stabilizer and dosage selection, compaction and curing methods, performance tests, and evaluation criteria. The survey results presented in the study are based on responses from participating highway agency and may not fully represent nationwide practices due to limited responses. Therefore, the results are interpreted as indicative trends rather than conclusive evidence and are considered alongside the literature review and SME interviews to support the experimental program.

### 5.1. Knowledge and Benefits of Using FDR

**Figure 4:**
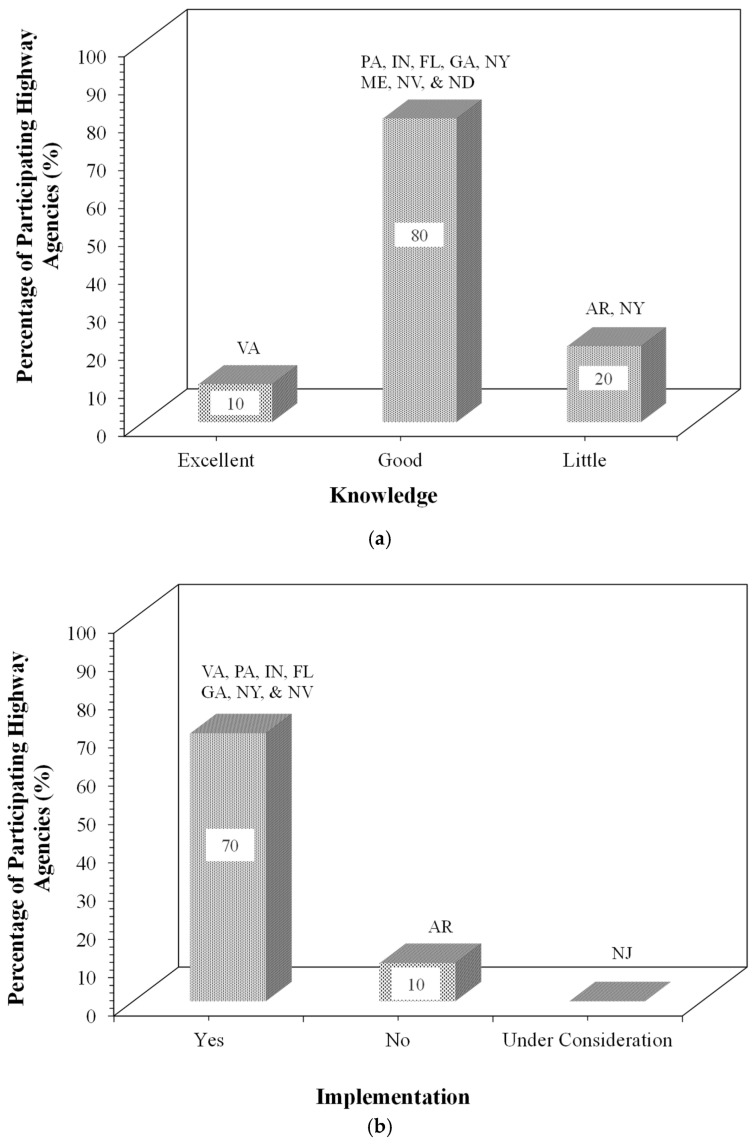
Use of FDR by participating highway state agencies: (**a**) knowledge and (**b**) implementation.

Participating agencies’ self-reported knowledge levels on FDR as “Little”, “Good”, and “Excellent,” and experience with implementation as “Yes”, “No”, or “Under consideration,” on an ordinal scale, reflected qualitative measurements rather than quantitative ones. Figure 4 illustrates highway agencies’ knowledge and experience with FDR implementation. Most responding agencies had at least a good knowledge and experience with the implementation of FDR (Figure 4). As shown in Figure 4, approximately 70% of the participating agencies reported implementing FDR, based on survey responses. NJ is shown separately and is not included in the percentage calculations. Virginia exhibited excellent knowledge and, therefore, adopted these techniques. Cost savings for FDR are between $10,000 and $30,000 per lane mile in Pennsylvania, and up to $50,000 per lane mile in Virginia and Indiana. Pennsylvania, Georgia, and Indiana selected FDR for its environmental and time savings benefits. New Jersey was not part of the survey responses and is shown separately for completeness; therefore, it is not included in the percentage calculations.

### 5.2. RAP Collection Methods for Mix Design

**Figure 5:**
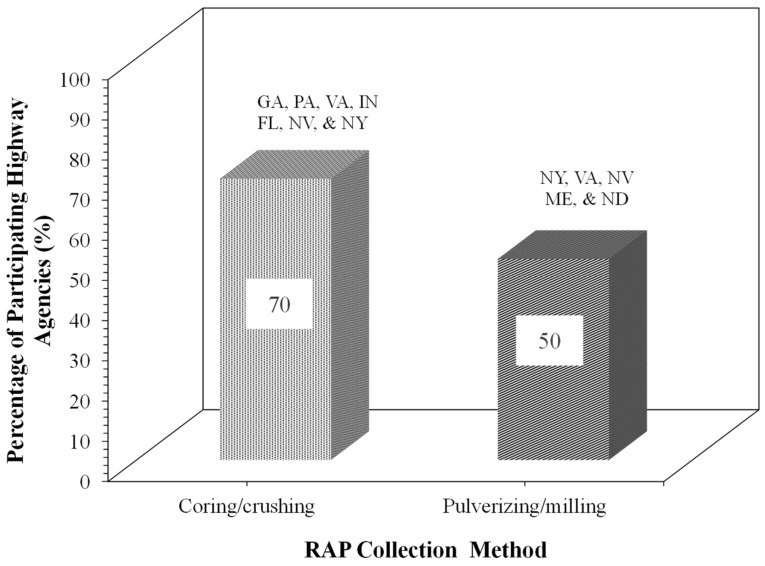
RAP collection methods reported by participating highway agencies for FDR mix design.

Figure 5 illustrates the RAP collection methods for FDR mix designs in the laboratory. RAP is collected mainly via coring (e.g., Pennsylvania, Georgia, Florida, Nevada, New York, Indiana, and Virginia) for FDR mix design (Figure 5). Crushing, reclaiming, and pulverization are also used in Virginia, Nevada, and Maine. The maximum size of RAP used in FDR mixtures varies between 1 in. and 3 in.

**Figure 6:**
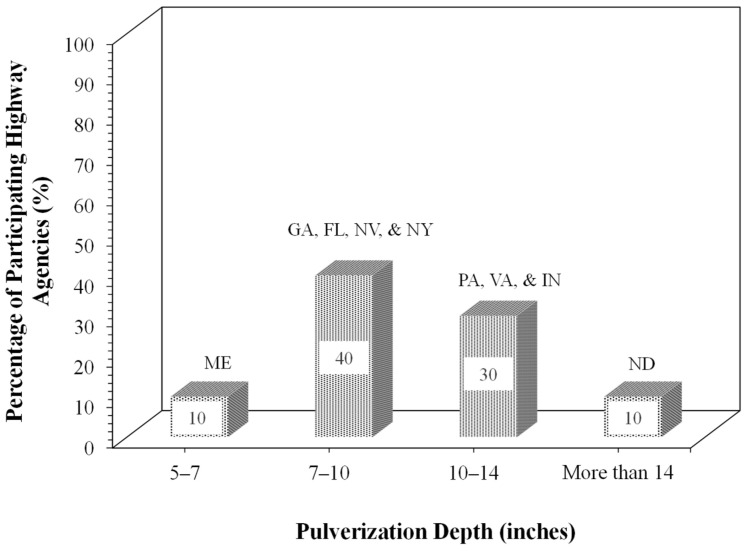
Pulverization depth used by participating highway agencies for FDR construction.

Figure 6 presents the pulverization depth for FDR construction. As can be seen from Figure 6, the depth of pulverization varied between 5 in. and 14 in. MDOT recommends a relatively lower pulverization depth (5–7 in.) compared to Pennsylvania and Virginia DOTs (10–14 in.).

### 5.3. Stabilizing Agents and Dosages

**Figure 7:**
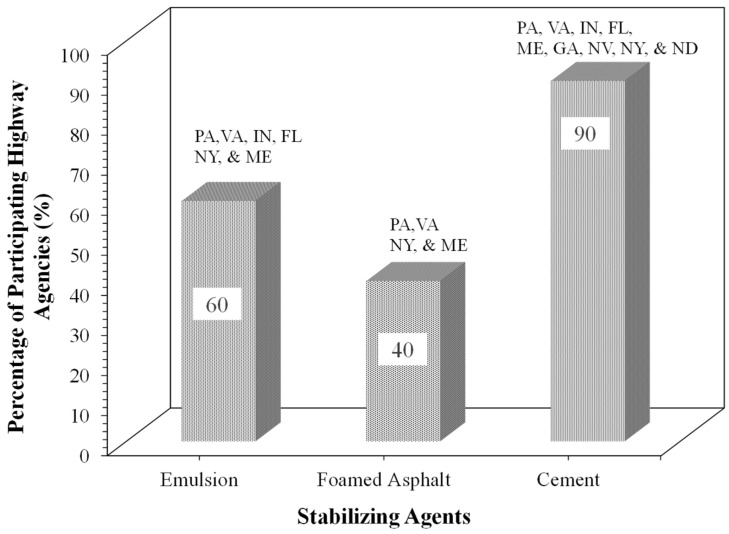
Stabilizing agents used for FDR mix design by participating highway agencies.

Figure 7 presents the stabilizing agents used for FDR mix design, as reported by the survey-responding states. As can be seen from Figure 7, cement is the most commonly used stabilizing agent as reported by 90% of participating agencies, while 60% of participating agencies use asphalt emulsion and 40% use foamed asphalt as the bituminous stabilizing agents. Georgia, Nevada, and North Dakota are some of the states that use only cement in their FDR mix design, while agencies in Pennsylvania, Virginia, New York, and Maine use both asphalt emulsion and foamed asphalt. For the agencies implementing FDR with only cement, the cement content ranges between 3% and 8%, by total mix weight. When bituminous additives are introduced to the FDR mixture, the typical cement contents range between 1% and 1.5% by total mix weight. Conversely, Indiana and Florida DOTs specify asphalt emulsion as the only bituminous additive, with contents of 3% and 2%, respectively. Foamed asphalt is added at 2–3% by total mix weight.

### 5.4. Compaction and Curing Process

**Figure 8:**
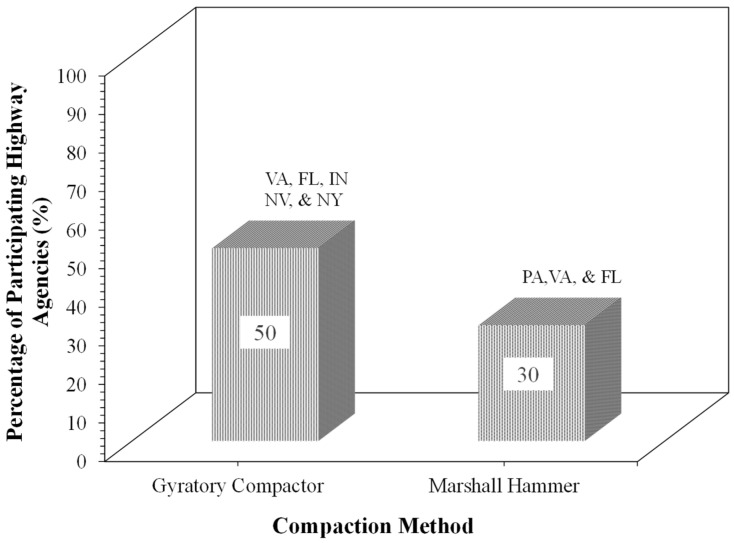
Compaction method for FDR mix design adopted by participating highway agencies.

Figure 8 illustrates the compaction method for FDR mix design. All the surveyed states use either the SGC with 30 gyrations (e.g., Indiana, Virginia, New York) or the Marshall method with 50 blows (e.g., Pennsylvania) or 75 blows (e.g., Virginia). Virginia and Florida use both methods, while Nevada does not perform any laboratory testing. In Virginia and Florida, foamed asphalt FDR specimens are cured at 40 °C to a constant mass, and FDR mixtures with asphalt emulsion are cured at 60 °C.

### 5.5. Performance Testing and Optimum Dosage Selection of Bituminous Additives

**Figure 9:**
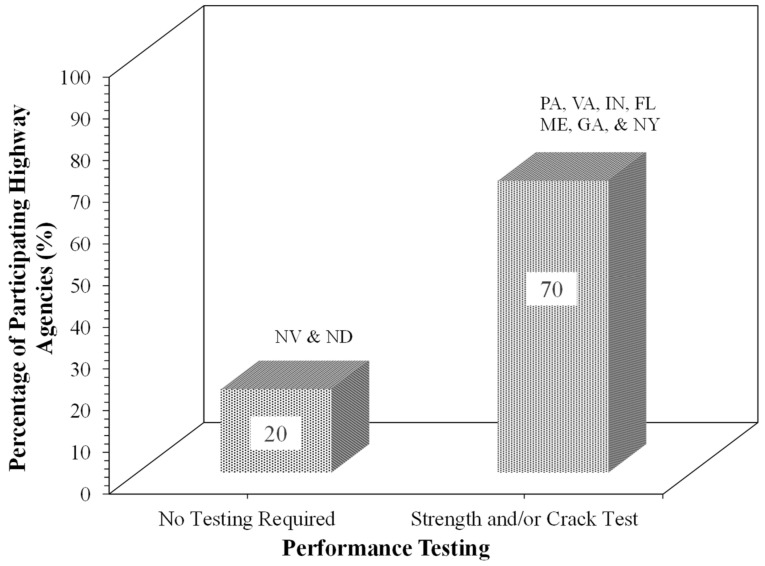
Performance testing methods adopted by participating highway agencies for FDR mixtures.

Figure 9 presents the performance testing methods for FDR mix design reported by the survey-responding agencies. As shown in Figure 9, 70% of participating highway agents adopted strength or crack tests to evaluate the performance of FDR mixtures, while a compressive strength test is also performed in several agencies, such as Indiana and Florida (Figure 9). Indiana also requires a crack test to be performed, while Florida performs the Marshall stability test.

### 5.6. Best Construction Practices

After compacting the FDR layer to the specified target density (typically between 95% and 98% based on laboratory compaction and field test strips), the in-place density, moisture content, thickness, and binder content are checked for quality control and acceptance. Pennsylvania an-d Indiana specify the maximum moisture content to be above 2%, while Georgia, Nevada, and Florida do not perform quality checks for moisture content. Curing time in the field varies greatly from one state to another, ranging from 1 day (Georgia) to more than 1 week (Pennsylvania). Pennsylvania, Virginia, and Indiana require a minimum 4 in. thick overlay, while Georgia requires 3–4 in.

**Figure 10:**
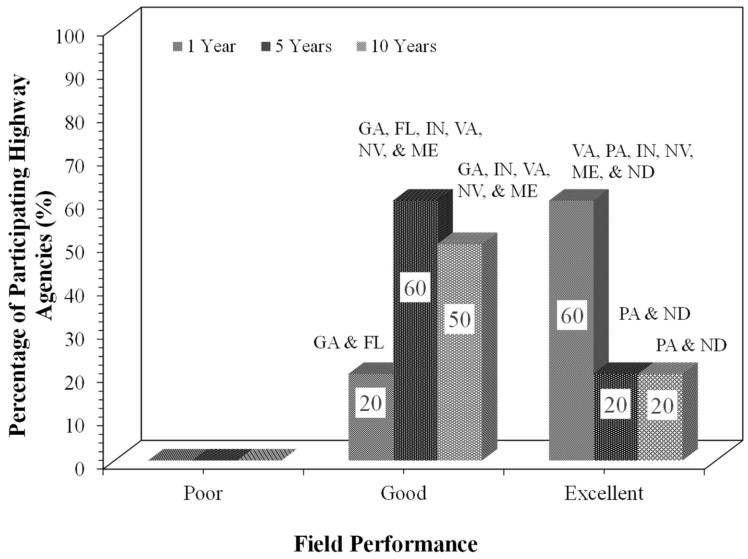
Reported field performance of FDR-rehabilitated pavements after 1, 5, and 10 years in service.

Figure 10 presents the field performance of FDR-rehabilitated pavements after one, five, and ten years in service. Participating highway agencies qualitatively rated the field performance of their FDR-rehabilitated pavements as “Poor”, “Good”, or “Excellent” based on observed surface conditions. An “Excellent” rating indicated no visible distress, “Good” indicated minor distress, and “Poor” indicated noticeable or structural distress. These ratings are based on highway agencies’ experiential observations rather than on quantitative performance indices. As can be seen from Figure 10, all the highway agencies that implemented FDR reported good performance one year after construction. Pennsylvania and North Dakota reported an excellent performance 10 years after construction.

### 5.7. Challenges and Recommendations

The major challenges encountered by highway agencies include identifying suitable project locations for FDR, managing traffic during half-width operations, ensuring adequate construction inspection and contractor experience, monitoring target density, early onset cracking due to excess cement content, maintaining consistent binder and water content, and addressing inconsistent historical records of as-builts. To overcome these challenges, the highway agencies (e.g., MDOT) suggest conducting an on-site review and verifying the existing pavement depth using cores, developing a better understanding of the mix design process, minimizing cement usage, and adopting a standardized curing process.

Overall, the survey results provide practical insights into current agency practices and support the literature synthesis presented in Section 4. These findings were used for the selection of stabilizer types, dosage ranges, compaction efforts, curing conditions, and performance tests adopted in the experimental program described in Section 7.

## 6. Best Practices from Literature, Survey Responses, and SME Interviews

The best practices summarized in this section are from the findings of the literature review, survey responses, and SME interviews to support the experimental program. These practices represent the commonly used approach for mix design and construction of FDR across the agencies. The following are identified as the best practices for FDR implementation based on documented experience and observed performance:

- The milling or coring method is commonly used to obtain RAP and soil particles for laboratory characterization and mix design. The pulverization depth for FDR typically ranges between 5 in. and 14 in. depending on the thickness of the existing asphalt layer and extent of pavement distresses.
- Asphalt emulsion content between 3% and 6%, and foamed asphalt between 2% and 3% are typical ranges commonly used to achieve better performance of FDR mixtures.
- Asphalt emulsion or foamed asphalt is generally combined with 1% to 1.5% of Portland cement to improve curing rate, gain early strength, and increase moisture resistance.
- Water content within a range from 2% to 3% is added to facilitate mixing and compaction of FDR mixtures and to enhance the hydration of Portland cement.
- FDR specimens are typically compacted using 30 gyrations of SGC or 75 Marshall blows for laboratory compaction. The SGC method is preferable as it provides consistent laboratory results and better field representation.
- FDR specimens with asphalt emulsion are cured at 60 °C for 72 h or to constant mass, and foamed asphalt mixtures are cured at 40 °C for 72 h or to constant mass. These conditions accelerate the curing process and develop maximum strength.
- The UCS test is widely adopted for cement-stabilized FDR mixtures as it effectively evaluates strength gain through hydraulic reactions. IDT is commonly used for bituminous-stabilized FDR mixtures to assess the cracking strength and moisture susceptibility. Additionally, APA is recommended by previous studies to determine the rutting susceptibility of cement- and bitumen-stabilized FDR mixtures.
- In the field, the FDR layer should be constructed when the ambient temperature is above 13 °C as low temperatures adversely affect mixing, compaction, strength gain, and long-term performance.
- A minimum of 95% maximum dry density is to be achieved when compacting the FDR layer.
- The FDR layer is cured based on two criteria: (1) moisture content below 2% and (2) a curing period of at least 3 days to ensure sufficient strength development prior to the placement of the overlay.
- In-place density, layer thickness, gradation, cement, and binder contents are measured to ensure the quality of the FDR layer.
- The FDR layer is generally topped with a hot mix asphalt (HMA) overlay (2–4 in.) of moderate thickness, with the overlay thickness determined based on anticipated traffic levels, to provide better riding quality and to protect the FDR layer from moisture-related damage.

## 7. Laboratory Performance Evaluation of FDR Mixtures with Bituminous and Cementitious Additives

The synthesis of the literature review, agency specifications, survey responses, and SME interviews identified major gaps, including variability in mix design practices across agencies and the limited consideration of rutting performance of FDR mixtures. Significant variability exists across the highway agencies in stabilizer type, dosage, water content, compaction, curing, performance tests, and performance criteria. To address these gaps, a laboratory testing program was developed based on the synthesis presented in Table 2 and Figure 4, Figure 5, Figure 6, Figure 7, Figure 8, Figure 9 and Figure 10. Three FDR mixtures: FDR-C, FDR-E, and FDR-F were selected to represent the commonly used cementitious and bituminous stabilization approaches. The laboratory performance of the mixtures was evaluated using the APA and IDT to access the rutting and cracking susceptibility, respectively. The following subsections discuss the materials, properties, the mix design procedure, and the performance testing results of the FDR mixtures.

### 7.1. Selection of Stabilizing Agents and Testing Parameters

The selection of stabilizing agents, dosage ranges, water content, compaction, curing, performance tests, and performance criteria for the laboratory testing was based on the synthesis presented in Table 2 and Figure 4, Figure 5, Figure 6, Figure 7, Figure 8, Figure 9 and Figure 10. As shown in Table 2 and Figure 7, Portland cement (Type I/II) was selected as the cementitious additive based on its adoption across the highway agencies, including Indiana, Georgia, South Carolina, Pennsylvania, and Virginia. Cement contents of 4%, 4.5%, and 5% by total weight of mix were selected for preparing FDR-C mixtures, consistent with the Indiana DOT recommendations (4–6%). A 0.5% increment was selected to capture the effect of small changes in cement content on strength and shrinkage cracking. Multiple studies [34,41,42] evaluated the same range of cement dosages for FDR-C mixtures and demonstrated that this range satisfied the strength requirements while limiting stiffness and shrinkage cracking. A maximum of 5% cement is considered in this study to minimize the risk of early onset of shrinkage cracking at higher cement contents.

Cationic slow-setting (CSS-1h) emulsion was selected for FDR-E mixtures due to widespread use and its compatibility with the aged binder, as recommended by the VDOT and PennDOT SMEs. The emulsion contents of 3%, 4%, and 5% by total mix weight were selected based on the typical range reported by the DOTs (Table 2; Figure 7). A minimum of 3% was selected to avoid raveling, while a maximum 5% was chosen to decrease the risk of bleeding [17,18]. Previous studies reported that FDR-E mixtures evaluated within this range improved rutting and cracking performance [8,13,25,42]. PG 64-22 foamed asphalt was selected for FDR-F mixtures based on PennDOT and VDOT recommendations (Table 2; Figure 7) and its favorable foaming characteristics including higher expansion ratio and longer half-life [35]. Although many DOTs specified foamed asphalt of 2–3% by total weight of mix, a wide range of 3–5% in 1% increments was selected to enable direct comparison with FDR-E mixtures at the same binder content and to evaluate the effect of higher foamed asphalt contents on the performance.

A constant cement of 1% by total mix weight was used in both FDR-E and FDR-F mixtures for early strength gain and moisture damage resistance [37], consistent with the 1–1.5% range reported by the DOTs (Table 2). A fixed water content of 3% by total mix weight was added to the FDR-E and FDR-F mixtures to isolate the effect of stabilizer type and dosage, in line with the PennDOT specifications (Table 2). A compaction effort of 30 SGC gyrations was selected based on its adoption by the majority of DOTs (Table 2; Figure 8) and its ability to represent field compaction [41]. A curing period of 1 week was selected for FDR-C mixtures based on the agency practices for cement-stabilized mixtures (Table 2; Figure 8). The FDR-E and FDR-F mixtures were cured at 60 °C for 3 days following the curing process recommended in a previous study [85], and PennDOT and VDOT specifications (Table 2). Both mixtures were cured under the same conditions to evaluate the effectiveness of bituminous additives.

The APA rut depth and ITS (Table 2) were used as performance measures to evaluate the rutting and cracking susceptibility of FDR mixtures and provide comparative analysis among the three mixture types. The APA test [57,58] was included to address a major gap in the current FDR practice, as no surveyed DOT specifies rutting criteria despite rutting being a primary distress mechanism in asphalt mixtures. The IDT (Figure 9) test was used to determine ITS of the three mixtures. Although IDT is not a cracking test, it is widely used as an indicator of mixture strength and durability, particularly for bitumen-stabilized FDR bases. The optimum dosage for each mixture was selected as the one minimizing the rut depth (lower than 10 mm) while maximizing ITS.

### 7.2. Materials and Properties

**Figure 11:**
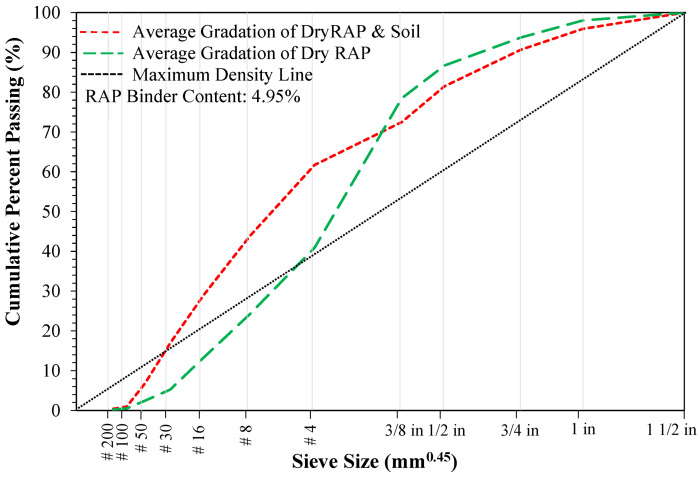
Properties of RAP and soil used in FDR mixtures.

RAP and soil were obtained by milling an existing pavement in Glassboro, New Jersey, USA and dried in an oven at 110 °F for a week; the gradations of RAP and soil were determined through sieve analyses in accordance with AASHTO T 27 [86]. In addition, the binder content of RAP was determined using extraction and recovery, and mineral matter tests in accordance with AASHTO T 319 [87] and AASHTO T 111 [88], respectively. Figure 11 presents the properties of RAP and soil used in FDR mixtures. From Figure 11, the nominal maximum aggregate size (NMAS) of RAP was 1 in., and RAP and soil presented considerably higher fines (by up to 30%) than RAP only. In addition, the binder content of RAP was determined as 4.95%. The NMAS of RAP falls within the 0.75–2 in. maximum particle size range specified by the majority of surveyed DOTs (Table 2) and satisfies ARRA’s requirement for 100% passing the 1.5 in. sieve [17,18,19,20].

#### Foaming Characteristics

Prior to preparing foamed asphalt mixtures, the foaming process was tested at different foaming water contents (2%, 2.5%, and 3% by total asphalt weight) and at different foaming temperatures (160 °C, 165 °C, and 170 °C) to determine the OFWC. In this study, a foaming machine was used to produce foamed asphalt by injecting cold water and air into hot asphalt (PG 64-22, based on the literature). In general, foamed asphalt binders with a low viscosity foam easily and present a higher expansion ratio and a longer half-life than those with high viscosity. The foaming test results showed that the increase in temperature from 160 °C to 165 °C increased the expansion ratio from 7 to 9 and the half-life from 8 to 9 s. However, the expansion ratio reduced from 9 to 8 and the half-life from 9 to 7 s, when the temperature increased from 165 °C to 170 °C. Results showed that at 165 °C, the foamed asphalt presented the best half-life of 9 s and an expansion ratio of 9. Therefore, the foaming process temperature selected for this study was 165 °C with a process water content of 2.5% of total asphalt weight.

### 7.3. Preparation of FDR Mixtures

**Figure 12:**
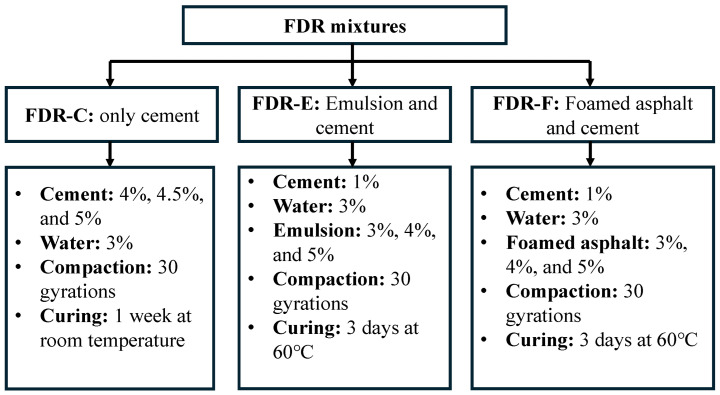
Experimental design for FDR mixtures with bituminous and cementitious additives.

To evaluate the impact of stabilizer type and dosage on the performance, all the mixtures were prepared by batching dry RAP and soil to the gradations presented in Figure 11, thereby eliminating gradation variability. Three FDR mixtures: FDR-C, FDR-E, and FDR-F were prepared with the dosages presented in Figure 12. For FDR-C, cement was added between 4% and 5% by total mix weight, in 0.5% increments, while asphalt emulsion and foamed asphalt of 3%, 4%, and 5% by total mix weight were selected for FDR-E and FDR-F, respectively.

RAP, water, and cement were mixed for 5 min in a mixer, after which the stabilizers were added at varying dosages and mixed for up to 8 min until the bituminous stabilizer was completely absorbed by the RAP. All FDR mixtures were then compacted at 30 gyrations using SGC. Once compacted, FDR-C specimens were allowed to cure at room temperature for 1 week, and FDR-E and FDR-F specimens at 60 °C for 3 days as shown in Figure 12.

After curing, air voids of FDR-E and FDR-F mixtures were determined using the CoreLok device (sourced from InstroTek Inc., Philadelphia, PA, USA) to assess mixture compactability and evaluate trends with increasing stabilizer dosage. As the air void level of FDR specimens is expected to be higher than that of typical asphalt mixtures, the CoreLok device is more suitable to measure both bulk specific gravity (Gmb; AASHTO T 331 [89]) and maximum theoretical specific gravity (Gmm; ASTM D6857 [90]) than the conventional method (AASHTO T 166 [91] and AASHTO T 209 [92], respectively), while for FDR-C, the air voids were excluded as measuring the Gmb was difficult due to falling rocks and weak bonding. Rutting susceptibility of all mixtures was evaluated using APA in accordance with AASHTO T340 [93], while cracking susceptibility was assessed by IDT following ASTM D6931 [94].

### 7.4. Performance Evaluation of FDR Mixtures

#### 7.4.1. Compaction Characteristics

**Figure 13:**
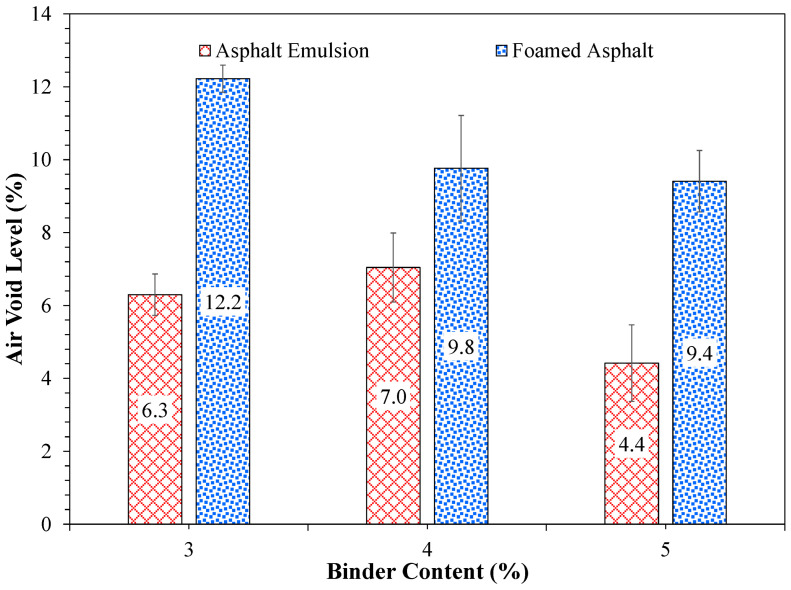
Air voids of FDR mixtures with asphalt emulsion and foamed asphalt.

Figure 13 summarizes the air void levels obtained for FDR mixtures at different binder contents. As can be seen from Figure 13, FDR-E presented a lower air void level than FDR-F by up to 5% at the same binder content. Additionally, FDR-E showed similar air void levels at 3% and 4% asphalt emulsion (within 1% air void). As the asphalt emulsion content increased to 5%, the air void level decreased to 4.4%. The higher air void levels in FDR-F mixtures may be attributed to their short half-life, which reduces the time available for binder dispersion and coating of aggregates during mixing. This traps air bubbles that are difficult to expel during compaction. In contrast, asphalt emulsion breaks gradually, allowing sufficient time for uniform distribution. In addition, water in the asphalt emulsion acts as a lubricant during compaction, allowing particle rearrangement and achieving better compaction and lower air voids in emulsion-stabilized FDR mixtures despite the lower residual binder content. The interpretations on foamed asphalt and asphalt emulsion are based on macroscopic observations in this study, and detailed microstructural and binder characterization would be required to fully explain these mechanisms. A detailed mechanistic understanding and characterization of foamed asphalt and asphalt emulsion would be required to fully explain their behavior. However, such detailed analyses were not included within the scope of the present study. These observations suggest that asphalt emulsion could be more efficient in filling the voids within the FDR mix than foamed asphalt.

#### 7.4.2. Rutting Susceptibility

**Figure 14:**
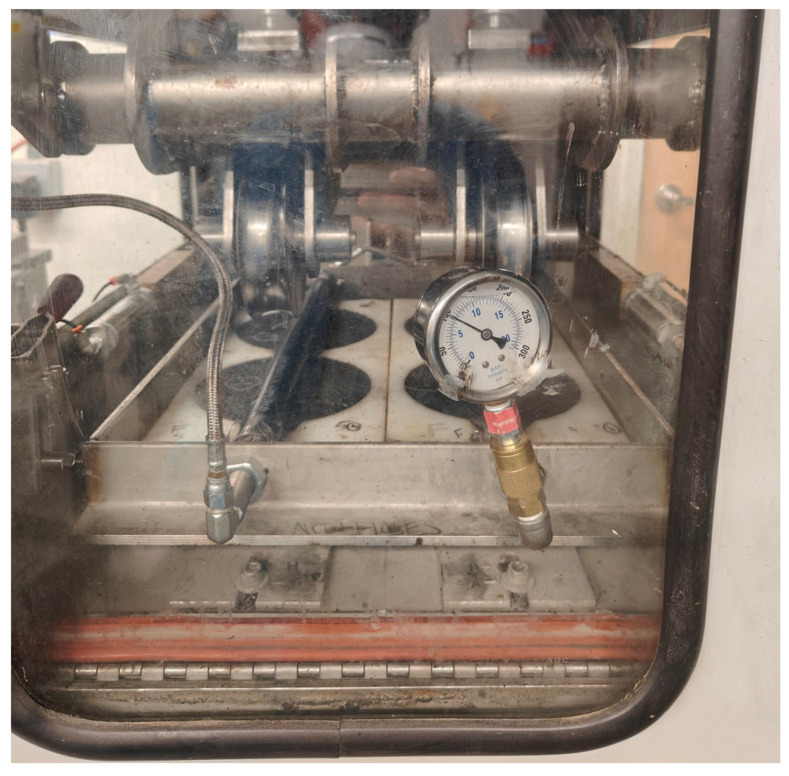
APA testing of FDR mixtures.

**Figure 15:**
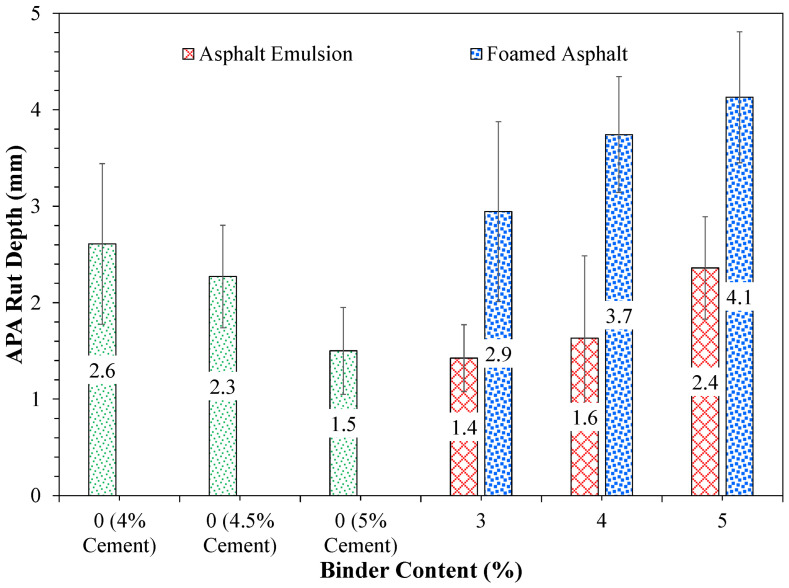
APA rut depth results of FDR mixtures with cement, asphalt emulsion, and foamed asphalt.

APA is a wheel-tracking device used for assessing the rutting susceptibility of hot-mix and cold-recycled asphalt mixtures under repeated loading. The present study considers the APA test to evaluate the rutting susceptibility of FDR mixtures based on the recommendations from previous studies by Saidi et al. and Cox et al. [57,78]. Figure 14 presents the APA testing of FDR mixtures. A load of 100 lbs. was applied to the specimens using steel wheels placed on top of pressurized hoses (100 ± 5 psi). Figure 15 illustrates the APA rut depth results for the three FDR mixtures after 8000 loading cycles. As can be seen from Figure 15, the rut depth increased with increasing bituminous additive content for both FDR-E and FDR-F. However, the rut depth decreased with the increase in cement content for FDR-C. This could be due to the increase in mixture stiffness and shear strength with increasing cement content, limiting shear flow. Among all the mixtures, FDR-E exhibited the lowest rut depth of 1.4 mm at 3% emulsion content, indicating higher rutting resistance. This can be attributed to the uniform distribution of asphalt emulsion and densification of mixture. FDR-C (at 4%, 4.5%, and 5% cement), FDR-E at all emulsion contents, and FDR-F at 3% foamed asphalt content presented rut depth values lower than 3 mm, which suggests that these FDR mixtures are better in resisting rutting.

Additionally, certain mixtures including FDR-C (at 4% and 4.5% cement), FDR-E (at 5% asphalt emulsion and 1% cement), and FDR-F (3% foamed asphalt and 1% cement) presented a similar rutting performance within a narrow range of 0.5 mm. Higher foamed asphalt contents (4% and 5%) caused an increase in the rut depth values of FDR-F by more than 2 mm compared to FDR-C at the highest cement content (5%). This suggests increasing the cement content to 5% for FDR-C or reducing the asphalt emulsion content to 3% for FDR-E reduces the rut depth considerably (by up to 80% for both mixtures). Overall, FDR mixtures at high cement contents (i.e., 5%) or those prepared with asphalt emulsion at 3% and at low cement contents (i.e., 1%) are the best at rutting.

#### 7.4.3. Cracking Potential

**Figure 16:**
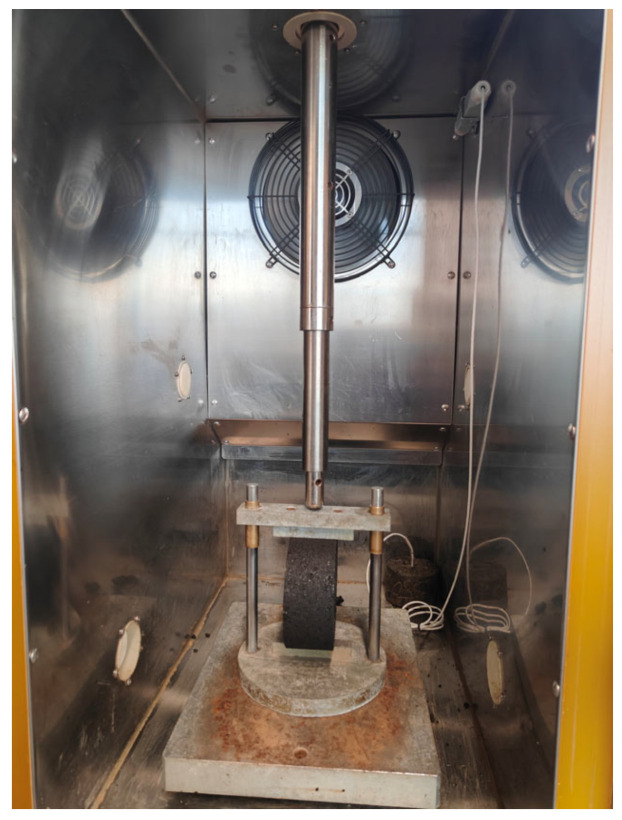
Indirect tensile (IDT) test of FDR mixtures.

**Figure 17:**
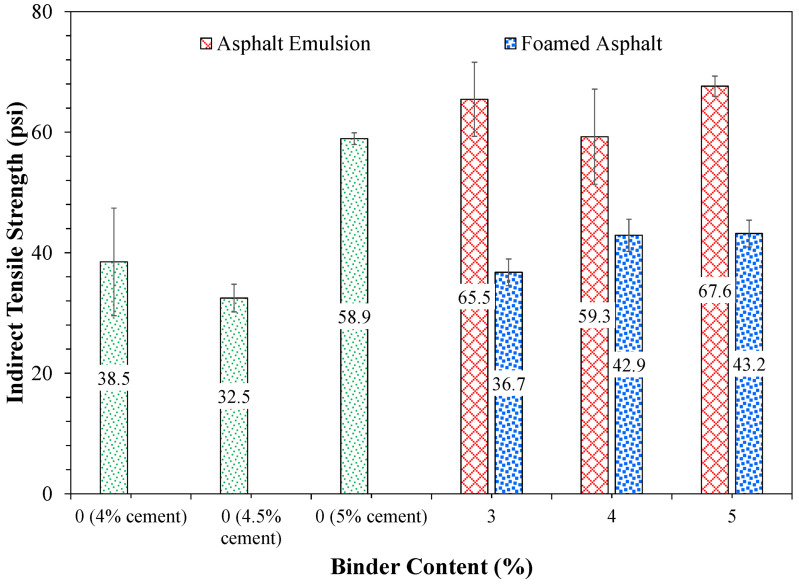
Indirect tensile strength results of the three FDR mixtures.

While the UCS test is more commonly used to characterize the strength of cement-stabilized FDR mixtures, the IDT test was adopted in this study for a consistent and comparative evaluation across cement-only and bitumen-stabilized FDR mixtures. The cracking potential of FDR mixtures was evaluated using IDT at 25 °C, in which FDR specimens were diametrically loaded at a rate of 50 mm/min until failure. Figure 16 presents the IDT test of FDR mixtures. The peak load at the failure of the specimen is measured to determine ITS of the specimen, as per Equation (1). Figure 17 illustrates ITS results for the three FDR mixtures. As can be seen from Figure 17, FDR-E at all asphalt emulsion contents and FDR-C at 5% cement presented higher ITS values than those of FDR-C at 4% and 4.5% cement and FDR-F at all foamed asphalt contents. This suggests that the addition of asphalt emulsion to FDR mixtures at low cement contents (i.e., 1%) improves the cracking resistance.

(1) $$ITS=\frac{2000}{\pi dt}$$

where

ITS—in kPa
P—applied load at failure, N
D—diameter of specimen, mm
t—thickness of specimen, mm

Additionally, increasing the cement content from 4.5% to 5% caused a considerable increase in ITS of FDR-C by over 50%, but lower than those of FDR-E at all binder contents. Conversely, FDR-C at 4% and 4.5%, and all FDR-F, presented similar ITS values (within 10 psi). This suggests that the addition of foamed asphalt does not impact the cracking resistance of FDR mixtures. The lower ITS values of FDR-F mixtures could be due to poor dispersion and coating efficiency of foamed asphalt with RAP and soil particles.

Overall, FDR-E at 3% and 5% asphalt emulsion presented the highest cracking resistance, followed by FDR-C at 5% cement. The higher ITS values of emulsion-stabilized FDR mixtures can be attributed to uniform coating of RAP and soil particles by asphalt emulsion, resulting in a continuous binder film that enhances cohesion and tensile strength. In contrast, cement-stabilized FDR mixtures attain strength through hydraulic reactions, but the existing asphalt binder (hydro-phobic) coating on RAP may reduce the cement–aggregate bonding by limiting hydration at the interface, resulting in comparatively lower tensile strength. These results suggest that asphalt emulsion with cement can improve the tensile strength of FDR mixtures and can be used for designing crack-resistant FDR mixtures.

Among the three mixtures, FDR-E with 3% asphalt emulsion exhibited the lowest APA rut depth and highest ITS value, maximizing both rutting and cracking resistance. Based on the outcomes of this study, a 3% asphalt emulsion with 1% cement is recommended to achieve optimal rutting and cracking resistance of FDR mixtures.

## 8. Conclusions and Recommendations

This study presented a comprehensive evaluation of full-depth reclamation (FDR) stabilization through the literature review, survey questionnaire responses, subject matter expert (SME) interviews, and laboratory testing of FDR mixtures with cement and bituminous additives at varying dosages. Although the limited number of respondents constrains the generalizability of the findings at the national level, the results offer valuable insights into emerging trends, mix design practices, and laboratory performance characteristics of FDR mixtures. The conclusions from the study are discussed below:

*Literature, survey questionnaire, and SME interviews*

- Portland cement (Type I/II) is the most commonly used stabilizing agent in FDR mixtures across the US. Cationic slow setting (CSS-1h) asphalt emulsion and performance grade (PG) 64-22 foamed asphalt are the widely used bituminous additives which perform better when compared to other bituminous additives. Among bituminous agents, asphalt emulsion is more employed than foamed asphalt.
- Cement-stabilized FDR mixtures with higher cement contents are susceptible to shrinkage cracking, which can propagate through the entire FDR layer, particularly in colder regions or in areas where reflective cracking is a concern.
- The dosage of asphalt emulsion and foamed asphalt varies between 1.5% and 3.5% depending on the design parameters considered for producing FDR. Portland cement is added with the bituminous-stabilized FDR mixtures to enhance the strength and to expedite the curing process.
- Superpave gyratory compaction (SGC) and the Marshall method are commonly used for compacting FDR mixtures. Two compaction efforts have been adopted by most state agencies: 30 gyrations using SGC and 75 Marshall blows per face. The Superpave gyratory compaction (SGC) method was observed to provide consistent laboratory compaction results and better representation of field compaction.
- The curing process of the recycled mixtures varied from one state to another. FDR mixtures with bituminous additives are mainly cured at 60 °C for 72 h to attain maximum strength. However, some agencies use 60 °C for 72 h for asphalt emulsion mixtures and 40 °C for 72 h for mixtures with foamed asphalt, while cement-stabilized mixtures are cured at room temperature for a week.
- Most highway agencies use strength-based tests such as unconfined compressive strength (UCS) for cement-stabilized and the indirect tensile (IDT) test for bituminous-stabilized FDR mixtures, with limited consideration of rutting performance.

*Laboratory Testing*

- FDR-E mixtures presented lower air void levels than those prepared with foamed asphalt by up to 4% at the same binder content, indicating better coating and compactability of emulsion. This can be attributed to the gradual breaking of emulsion during mixing, which enables uniform distribution of binder and allows moisture to act as a lubricant for compaction.
- Increasing the cement content from 4% to 5% enhanced the rutting resistance by 43% in FDR-C mixtures, while increasing the bituminous content from 3% to 5% increased the APA rut depth of both FDR-E and FDR-F mixtures. The decreased rut depth in FDR-C could be due to the increased stiffness.
- Increasing the cement content from 4% to 5% increased the cracking resistance by 53% in FDR-C mixtures.
- FDR-E with 3% asphalt emulsion and 1% cement reported the lowest Asphalt Pavement Analyzer (APA) rut depth (1.4 mm) and the highest indirect tensile strength (ITS) among all the mixture types and dosage levels tested, optimizing both rutting and cracking resistance.
- FDR-E with 3% asphalt emulsion and 1% cement improved the rutting performance by approximately 46% and the cracking performance by 70% compared to the control FDR-C with 4% cement.
- Foamed asphalt did not improve the rutting or cracking performance relative to cement-only and asphalt emulsion-stabilized FDR mixtures. The APA rut depths of FDR-F at 4% and 5% foamed asphalt were 2 mm more than FDR-C at 5% and the ITS values were comparable to FDR-C at 4% and 4.5%. The poor performance of FDR-F could be attributed to the rapid foam collapse during mixing, resulting in non-uniform binder dispersion.
- The Asphalt Pavement Analyzer (APA) and indirect tensile (IDT) tests were shown to be effective for comparing rutting and cracking susceptibility of the cement-only and bituminous-stabilized FDR mixtures.

Based on the findings from the literature, survey questionnaire responses, SME interviews, and laboratory testing results, the following recommendations guide FDR mix design and construction practices and future research:

- Given the heterogeneous and complex nature of FDR mixtures, a performance-based mix design approach allows more rational determination of stabilizer dosages compared to conventional methods that rely on fixed threshold limits from individual standard tests.
- Asphalt emulsion combined with cement exhibited better performance in terms of rutting and cracking resistance compared to the cement-only and foamed asphalt stabilized FDR mixtures. Accordingly, asphalt emulsion-stabilized FDR mixtures can be a viable option, particularly where cracking is a concern.
- The Asphalt Pavement Analyzer (APA) test can be used to assess the rutting performance of cement- and bitumen-stabilized FDR mixtures.
- Field performance of FDR is significantly influenced by cement dosage, binder content, moisture content, pulverization depth, compaction, and curing period. Hence, in-place density, pulverization depth, moisture, and stabilizing agents’ dosage control is essential to ensure long-term performance and durability of FDR pavements.
- This study recommends additional research on laboratory testing methods to evaluate the performance and durability of FDR mixtures, as well as full-scale testing to support the development of standardized specifications for broader implementation of FDR.

Overall, the primary contribution of the study is the demonstration of a performance-based approach for evaluating FDR mixtures that balances both rutting and cracking resistance. The findings support a performance-based mix design approach for FDR mixtures with bituminous and cementitious additives and provide guidance for future research. However, fatigue performance and durability aspects (e.g., moisture susceptibility), as well as binder characterization and microstructural analysis, were not included in this study and are recommended for future research.

## Figures and Tables

**Table 1:** Comparison of stabilizing agents of FDR mixtures.

Stabilizing Agent	Advantages	Disadvantages	Application
Cement [34,41,42]	Rapid strength gain, high stiffness, and high rutting and moisture resistance.	Brittle behavior, prone to shrinkage cracking, and sensitive to water content.	Heavy traffic loads and freeze–thaw regions.
Lime [43]	Cost-effective.	Low early strength, longer curing, and sensitive to water content.	Low traffic loads and cost-sensitive projects.
Fly-ash [44]	Cost-effective and strength gain.	Leaching potential, lower early strength, and longer curing.	Soil stabilization.
Asphalt emulsion [8,13,25,42]	Low construction temperature, compatible with RAP and soil materials, and less carbon emissions.	Low early strength, sensitive to water content, and poor fatigue resistance.	Rural highways and high RAP content pavements.
Foamed asphalt [28,38]	Low construction temperature, rapid curing, and moisture resistance.	Temperature dependent, rapid stiffness development, and complex equipment.	Low-volume roads and moisture-prone regions.

**Table 2:** Summary of specifications of highway agencies for FDR.

State DOTs	RAP Size (Maximum Particle Size)	Stabilizing Agents (by Weight of Mixture)	Water	Compaction	Curing	Selection of Optimum Contents
Indiana [69,70]	2 in.	Asphalt emulsion Portland cement (4–6%)	Moisture density curve	30 gyrations	40 °C for 72 h 25 °C for 1 week	ITS UCS
California [28,71]	2 in.	Foamed asphalt	Moisture density curve	Hveem method	38 °C for 72 h	ITS dry, wet and TSR
Pennsylvania [22]	2 in.	Cationic emulsion (3–6%) Foamed asphalt: PG 64-22, PG 58-22, or PG 58-28 (2–3.5%)	3% or moisture density curve	4 in. or 6 in. height	40 °C for 72 h	ITS
Texas [72,73,74]	0.75 in.	Asphalt emulsion (CSS-1h) (3–5%) Foamed asphalt (2–3%)	Moisture density curve	30 gyrations	40 °C for 72 h	ITS
Virginia [23]	2 in.	Asphalt emulsion (3.0%) Foamed asphalt (2.5%)	2% or moisture density curve	30 gyrations or 75 Marshall blows	60 °C to constant mass 40 °C for 72 h	Marshall stability dry ITS
Florida [75]	2 in.	Asphalt emulsion	Moisture density curve	30 gyrations or 50 Marshall blows	60 °C to constant mass	Wet Marshall stability about 70% of dry
Minnesota [76]	1.5 in.	Asphalt emulsion (CSS-1h)	Moisture density curve	Not available	Not available	Not available
Illinois [77,78]	1.5 in.	Asphalt emulsion Foamed asphalt	Moisture density curve	30 gyrations or 75 Marshall blows	Not available	ITS
South Carolina [79,80]	1.75 in.	Asphalt emulsion (3–6%) Portland cement (3%, 6%, and 9%)	Moisture density curve	Standard Proctor hammer with 3 layers 25 blows each 30 gyrations	60 °C for 48 h 25 °C for 1 week	Marshall stability, flow, and ITS UCS
Georgia [81,82]	0.08 in.	Portland cement (3%, 6%, 7%, and 9%)	Moisture density curve	Standard Proctor hammer with 3 layers 25 blows each	25 °C for 1 week	UCS

## Data Availability

The original contributions presented in this study are included in the article/Supplementary Materials. Further inquiries can be directed to the corresponding author.

## Supplementary Materials

The following supporting information can be downloaded at: https://www.mdpi.com/article/10.3390/ma19081540/s1.

## Abbreviations

The following abbreviations are used in this manuscript:

FDR	Full-depth reclamation
SMEs	Subject matter experts
APA	Asphalt Pavement Analyzer
IDT	Indirect tensile
CCPR	Cold central plant recycling
ARRA	Asphalt Recycling and Reclaiming Association
NCHRP	National Cooperative Highway Research Program
DOT	Department of transportation
FDR-C	Cement-only
FDR-E	Asphalt emulsion and cement
FDR-F	Foamed asphalt and cement
VDOT	Virginia department of transportation
PennDOT	Pennsylvania department of transportation
IDOT	Illinois department of transportation
TDOT	Texas department of transportation
CDOT	Colorado department of transportation
SCDOT	South Carolina department of transportation
NJDOT	New Jersey department of transportation
NYDOT	New York department of transportation
NDOT	Nevada department of transportation
AASHTO	American Association of State Highway and Transportation Officials
RAP	Reclaimed asphalt pavement
PCA	Portland cement association
CSS-1h	Cationic slow setting
HFMS-2	High-float medium-setting with a solvent
HFMS-2p	High-float medium-setting modified with a polymer
OFWC	Optimum foaming water content
OWC	Optimum water content
SGC	Superpave gyratory compactor
M_r_	Resilient modulus
E*	Complex modulus
UCS	Unconfined compressive strength
ITS	Indirect tensile strength
TSR	Tensile strength ratio
OBC	Optimum binder content
INDOT	Indiana department of transportation
FDOT	Florida department of transportation
MDOT	Maine department of transportation
NMAS	Nominal maximum aggregate size
PG	Performance grade

## References

[1] Iwański M., Mazurek G., Buczyński P., Iwański M.M. (2022). Effects of Hydraulic Binder Composition on the Rheological Characteristics of Recycled Mixtures with Foamed Bitumen for Full Depth Reclamation. Constr. Build. Mater..

[2] Afonso M.L., Dinis-Almeida M., Pereira-de-Oliveira L.A., Castro-Gomes J., Zoorob S.E. (2016). Development of a Semi-Flexible Heavy Duty Pavement Surfacing Incorporating Recycled and Waste Aggregates—Preliminary Study. Constr. Build. Mater..

[3] Xiao F., Yao S., Wang J., Li X., Amirkhanian S. (2018). A Literature Review on Cold Recycling Technology of Asphalt Pavement. Constr. Build. Mater..

[4] Lewis D.E., Jared D.M., Torres H., Mathews M. (2006). Georgia’s Use of Cement-Stabilized Reclaimed Base in Full-Depth Reclamation. Transp. Res. Rec..

[5] Le J.L., Marasteanu M., Milavitz R. (2016). Investigation of Performance Requirements of Full-Depth Reclamation Stabilization.

[6] Jones D., Louw S., Wu R. (2016). Full-Depth Reclamation: Cost-Effective Rehabilitation Strategy for Low-Volume Roads. Transp. Res. Rec. J. Transp. Res. Board.

[7] Ghanizadeh A.R., Rahrovan M., Bafghi K.B. (2018). The Effect of Cement and Reclaimed Asphalt Pavement on the Mechanical Properties of Stabilized Base via Full-Depth Reclamation. Constr. Build. Mater..

[8] Alizadeh A., Modarres A. (2019). Mechanical and Microstructural Study of RAP–Clay Composites Containing Bitumen Emulsion and Lime. J. Mater. Civ. Eng..

[9] Torres-Machi C., Evers E., Schmidt J., Crayton A. (2024). Pavement Rehabilitation Analysis: A Life-Cycle Cost and Long-Term Performance Comparison of Full Depth Reclamation and Overlays.

[10] Thakur A., Ashish P.K., Das D., Misra S. (2026). Is Full Depth Reclamation Always a Sustainable Alternative to Traditional Reconstruction? Context Dependent Shifts in Making Sustainable Rehabilitation Choice. Environ. Impact Assess. Rev..

[11] Zhang H., Zhang Q., Luo C., Liu N., Wang K. (2025). Life Cycle Assessment and Sensitivity Analysis of Carbon Emissions in Full Depth Reclamation with Portland Cement and Conventional Pavement Repair. Sci. Rep..

[12] Asphalt Recycling and Reclaiming Association (ARRA) (2015). Basic Asphalt Recycling Manual.

[13] (2020). Asphalt Academy Bitumen Stabilized Materials: A Guide for the Design and Construction of Bitumen Emulsion and Foamed Bitumen Stabilized Materials.

[14] Henrichs C. (2015). Laboratory Comparison of Full Depth Reclamation Stabilization Laboratory Comparison of Full Depth Reclamation Stabilization Techniques Using Arkansas Field Materials Techniques Using Arkansas Field Materials. Master’s Thesis.

[15] Bemanian S., Polish P., Maurer G. (2006). Cold In-Place Recycling and Full-Depth Reclamation Projects by Nevada Department of Transportation. Transp. Res. Rec. J. Transp. Res. Board.

[16] National Cooperative Highway Research Program (NCHRP) Synthesis 657. Full-Depth Reclamation: Current Practices (2025). Transportation Research Board 2025. https://www.nationalacademies.org/publications/29222.

[17] Asphalt Recycling and Reclaiming Association (ARRA) (2023). Recommended Mix Design Guidelines for Full Depth Reclamation (FDR) Using Emulsified Asphalt Stabilizing Agent FDR201A. https://cdn.ymaws.com/www.arra.org/resource/resmgr/guidelines/march_2023_updates/arra_fdr201a_1-19-23.pdf.

[18] Asphalt Recycling and Reclaiming Association (ARRA) (2017). Recommended Construction Guidelines for Full Depth Reclamation (FDR) Using Bituminous Stabilization FDR101. https://cdn.ymaws.com/www.arra.org/resource/resmgr/Guidelines/ARRA_FDR101_11-02-17.pdf.

[19] Asphalt Recycling and Reclaiming Association (ARRA) (2017). Recommended Construction Guidelines for Full Depth Reclamation (FDR) Using Cementitious Stabilization FDR102. https://cdn.ymaws.com/www.arra.org/resource/resmgr/Guidelines/ARRA_FDR102_09-12-17.pdf.

[20] Asphalt Recycling and Reclaiming Association (ARRA) (2021). Recommended Mix Design Guidelines for Full Depth Reclamation (FDR) Using Cement or Cement Kiln Dust (CKD) Stabilizing Agent FDR202. https://cdn.ymaws.com/www.arra.org/resource/resmgr/guidelines/jan_2023_updates/arra_fdr202_8-26-21.pdf.

[21] (2022). Standard Practice for Emulsified Asphalt Content of Full-Depth Reclamation Mixture Design.

[22] Pennsylvania Department of Transportation (2020). Publication 408/2020 Specifications.

[23] Virginia Department of Transportation (2016). 2016 Road and Bridge Specifications: Division III Roadway Construction, Special Provision Copied Notes (SPCNs), Special Provisions (SPs), and Supplemental Specifications (SSs).

[24] Maine Department of Transportation (MaineDOT) (2020). Standard Details.

[25] Shuler S. (2015). Best Practices for Full-Depth Reclamation Using Asphalt Emulsions.

[26] Reeder G.D., Harrington D.S., Ayers M.E., Adaska W. (2017). Guide to Full-Depth Reclamation (FDR) with Cement. http://www2.cement.org/pdf_files/sr1006p.pdf.

[27] Daniel E.W., Mohammadreza S., Joe K., Renae K. (2017). Base Stabilization Guidance and Additive Selection for Pavement Design and Rehabilitation. https://rosap.ntl.bts.gov/view/dot/35083.

[28] Jones D., Fu P., Harvey J.T. (2009). Full-Depth Pavement Reclamation with Foamed Asphalt in California: Guidelines for Project Selection, Design, and Construction.

[29] Fedrigo W., Núñez W.P., Kleinert T.R., Matuella M.F., Ceratti J.A.P. (2017). Strength, Shrinkage, Erodibility and Capillary Flow Characteristics of Cement-Treated Recycled Pavement Materials. Int. J. Pavement Res. Technol..

[30] (1992). Soil-Cement Laboratory Handbook. Portland Cement Association. https://secement.org/wp-content/uploads/2017/04/EB052.07s.pdf.

[31] Ghasemi P., Yu J.L.P., Williams R.C., Jahren C. (2018). Field Investigation of Stabilized Full-Depth Reclamation (SFDR). https://rosap.ntl.bts.gov/view/dot/64619.

[32] Li Y., Luo C., Ji K., Zhang H., Sun B. (2020). Laboratory evaluation of strength performance of Full-Depth reclamation with Portland cement material. Coatings.

[33] Xia Q., Zhang H., Miao W., Guo X., Zhang Q. (2025). Multi-objective optimization of full depth reclamation with Portland cement using NSGA-II for sustainable pavement rehabilitation. Front. Built Environ..

[34] Fedrigo W., Núñez W.P., Visser A.T. (2020). A review of full-depth reclamation of pavements with Portland cement: Brazil and abroad. Constr. Build. Mater..

[35] Morian D.A., Solaimanian M., Scheetz B., Jahangirnejad S. (2012). Developing Standards and Specifications for Full Depth Pavement Reclamation.

[36] Casillas S., Braham A.F. (2022). Development of a Performance-Based Approach to Asphalt Emulsion Selection for Cold In-Place Recycling Applications. Transp. Res. Rec. J. Transp. Res. Board.

[37] Zhu J., Ma T., Fang Z. (2020). Characterization of Agglomeration of Reclaimed Asphalt Pavement for Cold Recycling. Constr. Build. Mater..

[38] Kuna K., Airey G., Thom N. (2014). Laboratory Mix Design Procedure for Foamed Bitumen Mixtures. Transp. Res. Rec. J. Transp. Res. Board.

[39] Texas Department of Transportation (2004). Special Specification 3254: Cold In-Place Recycling of Asphalt Concrete Pavement. CSJ: 0425-01-019, 0030-05-062 & 0425-01-019. https://ftp.txdot.gov/pub/txdot-info/cmd/cserve/specs/1993/spec/es3254.pdf.

[40] Mallick R.B., Teto M.R., Kandhal P.S., Ray Brown E., Bradbury R.L., Kearney E.J. (2002). Laboratory Study of Full-Depth Reclamation Mixes. Transp. Res. Rec..

[41] Ogundipe T., Braham A., Tingle J.S. (2025). Assessing the Effect of Material Properties on Full-Depth Reclamation Mixtures via Compressive Strength Characterization. Transp. Res. Rec. J. Transp. Res. Board.

[42] Sahayogi K.A. (2025). Consideration and Preparation of Job Mix Formula for Full Depth Reclamation Pavement by Chemical Stabilization. Int. J. Appl. Math..

[43] Chkolyan K. (2025). Application of Basalt Fibers and Cement Through FDR Technology in the km 93 + 880 Section of the M2 Yerevan–Goris–Meghri Highway. J. Archit. Eng. Res..

[44] Wolfe W., Butalia T.S., Walker H. Full-depth reclamation of asphalt pavements using lime-activated Class F fly ash: Structural monitoring aspects. Proceedings of the 2009 World Coal of Ash Conference.

[45] Anderson D.A., Luhr D.R., Lahr M. (1985). Cold In-Place Recycling of Low-Volume Roads in Susquehanna County Volume I: Technical Report.

[46] (2020). Standard Method of Test for Moisture–Density Relations of Soil-Cement Mixtures.

[47] (2020). Standard Method of Test for Moisture–Density Relations of Soils Using a 4.54-kg Rammer and a 457-mm Drop.

[48] New York State Department of Transportation (2015). Design and Construction Guidelines for Full Depth Reclamation of Asphalt Pavement. Geotechnical Engineering Manual GEM-27. https://www.dot.ny.gov/divisions/engineering/technical-services/technical-services-repository/GEM-27b.pdf.

[49] Mallick R.B., Kandhal P.S., Ray E., Bradbury R.L., Kearney E.J. (2002). Development of a Rational and Practical Mix Design System for Full Depth Reclaimed (FDR) Mixes.

[50] Kim Y., Lee H.D., Heitzman M. (2007). Validation of New Mix Design Procedure for Cold In-Place Recycling with Foamed Asphalt. J. Mater. Civ. Eng..

[51] Arambula-Mercado E., Sebesta S., Kim J., Taylor R. (2020). Evaluation of Mix Design Variables for Full Depth Reclamation (FDR) with Asphalt Unclassified. https://static.tti.tamu.edu/tti.tamu.edu/documents/0-7076-R1.pdf.

[52] Cardone F., Grilli A., Bocci M., Graziani A. (2015). Curing and Temperature Sensitivity of Cement–Bitumen Treated Materials. Int. J. Pavement Eng..

[53] Tebaldi G., Dave E.V., Marsac P., Muraya P., Hugener M., Pasetto M., Graziani A., Grilli A., Bocci M., Marradi A. (2014). Synthesis of Standards and Procedures for Specimen Preparation and In-Field Evaluation of Cold-Recycled Asphalt Mixtures. Road Mater. Pavement Des..

[54] Ben Yahya M., Lamothe S., Lachance-Tremblay É. (2025). Effect of Temperature and Relative Humidity on Cement-Bitumen Treated Materials Produced in the Laboratory Using Emulsion or Foamed Bitumen. Constr. Build. Mater..

[55] Marquis B., Bradbury R.L., Colson S., Graduate Y.V.N., Assistant R., Gould J.S., O’brien S. Design, Construction and Early Performance of Foamed Asphalt Full Depth Reclaimed (FDR) Pavement in Maine. Proceedings of the TRB 82nd Annual Meeting.

[56] Thompson M.R., Garcia L., Carpenter S.H. (2009). Cold In-Place and Full-Depth Recycling with Asphalt Products (CIR&FDRwAP).

[57] Silva A.H.M., Vasconcelos K.L., Aranha A.L., Bernucci L.B., Chaves J.M. (2013). Laboratory and Field Evaluation of Cold-in-Place RAP Recycling.

[58] Charmot S., Braham A., Zheng K. (2013). Effect of Emulsion Content and Cement Loading on Cold Recycling Mixture Fracture Energy Measured Using Semicircular Bending Fracture Test.

[59] Euch Khay S.E., Euch Ben Said S.E., Loulizi A., Neji J. (2015). Laboratory Investigation of Cement-Treated Reclaimed Asphalt Pavement Material. J. Mater. Civ. Eng..

[60] Wang T., Xiao F., Zhu X., Huang B., Wang J., Amirkhanian S. (2018). Energy Consumption and Environmental Impact of Rubberized Asphalt Pavement. J. Clean. Prod..

[61] Sun H., Zhang X., Hu Z., Liu Y., Yang Z. (2026). Performance Evaluation of Polymer Latex-Modified Asphalt Emulsion and Cold Recycling Mixture. Constr. Build. Mater..

[62] Kim Y., Lee H.D. (2006). Development of Mix Design Procedure for Cold In-Place Recycling with Foamed Asphalt. J. Mater. Civ. Eng..

[63] Kim Y., Im S., Lee H.D. (2011). Impacts of Curing Time and Moisture Content on Engineering Properties of Cold In-Place Recycling Mixtures Using Foamed or Emulsified Asphalt. J. Mater. Civ. Eng..

[64] Lee K.W.W., Mueller M., Singh A. (2014). Cold In-Place Recycling as a Sustainable Pavement Practice. J. Civ. Eng. Archit..

[65] Ozer H. (2015). Applications of Performance-Based Specifications for Mix Designs. https://ws.engr.illinois.edu/sitemanager/getfile.asp?id=1252.

[66] Cox B.C., Howard I.L., Chesner W.H. (2015). Cold In-Place Recycling Characterization Framework and Design Guidance for Single or Multiple Component Binder Systems. No. FHWA/MS-DOT-RD-15-250-Volume2. http://www.cee.msstate.edu/publications/2015_FHWA_MS_DOT_RD_15_250_Vol_2_CIR.pdf.

[67] Cross S.A. (1999). Experimental Cold In-Place Recycling with Hydrated Lime. Transp. Res. Rec. J. Transp. Res. Board.

[68] Owino J., Onyango M., Wu W., Fomunung I., Brown H.J., Dumbiri O., Msechu K. (2022). Performance Evaluation of Full Depth Reclaimed (FDR) Pavements in Tennessee (No. RES2020-11). https://rosap.ntl.bts.gov/view/dot/62733.

[69] Indiana Department of Transportation (2020). Mix Design Procedure for Full Depth Reclamation (FDR) with Asphalt Emulsion.

[70] Indiana Department of Transportation (2022). Mix Design Procedure for Full Depth Reclamation (FDR) with Cement.

[71] California Department of Transportation (2018). Standard Specifications.

[72] Texas Department of Transportation (2024). Full-Depth Reclamation with Asphalt Binders Construction Guidelines Materials and Tests.

[73] Texas Department of Transportation (2020). Special Specification 3089 Full Depth Reclamation Using Asphalt Emulsion (Road-Mixed).

[74] Texas Department of Transportation (2020). Special Specification 3088 Full Depth Reclamation Using Foamed Asphalt (Road-Mixed).

[75] Florida Department of Transportation (2024). Section 332: Full Depth Reclamation.

[76] Minnesota Department of Transportation (2018). Full Depth Reclamation Reference Guide: Special Provision.

[77] Illinois Department of Transportation (2019). Special Provision for Full-Depth Reclamation (FDR) with Emulsified Asphalt.

[78] Illinois Department of Transportation (2019). Special Provision for Full-Depth Reclamation (FDR) with Foamed Asphalt.

[79] South Carolina Department of Transportation (2017). Standard Method of Test for Sampling, Preparing and Testing of Cement Modified Recycled Base Compression Specimens in the Laboratory SCDOT Designation: SCT-26 (08/2017).

[80] South Carolina Department of Transportation (2008). Standard Method of Test for Full Depth Reclamation Using Asphalt Emulsion—Job Mix Formula Preparation SCDOT Designation: SC-T-99 (7/08).

[81] Georgia Department of Transportation (2018). Standard Specification: Section 315—Cement Stabilized Reclaimed Base Construction (CSRB).

[82] Georgia Department of Transportation (2018). GDT 65: Laboratory Design of Soil-Cement and Cement Stabilized Graded Aggregate.

[83] (1990). National Cooperative Highway Research Program (NCHRP) Synthesis of Highway Practice 160: Cold-Recycled Bituminous Concrete Using Bituminous Materials. https://onlinepubs.trb.org/Onlinepubs/nchrp/nchrp_syn_160.pdf.

[84] National Cooperative Highway Research Program (NCHRP) Synthesis 421 (2011). Recycling and Reclamation of Asphalt Pavements Using In-Place Methods.

[85] Saidi A., Ali A., Lein W., Mehta Y. (2019). A Balanced Mix Design Method for Selecting the Optimum Binder Content of Cold In-Place Recycling Asphalt Mixtures. Transp. Res. Rec..

[86] (2023). Standard Method of Test for Sieve Analysis of Fine and Coarse Aggregates.

[87] (2022). Standard Method of Test for Quantitative Extraction of Asphalt Binder from Asphalt Mixtures.

[88] (2022). Standard Method of Test for Mineral Matter or Ash in Asphalt Materials.

[89] (2023). Standard Method of Test for Bulk Specific Gravity (Gmb) and Density of Compacted Asphalt Mixtures Using Automatic Vacuum Sealing Method (ASTM Designation: D6752/D6752M-18).

[90] (2021). Standard Test Method for Maximum Specific Gravity and Density of Asphalt Mixtures Using Automatic Vacuum Sealing Method.

[91] (2022). Standard Method of Test for Bulk Specific Gravity (Gmb) of Compacted Asphalt Mixtures Using Saturated Surface-Dry Specimens.

[92] (2023). Standard Method of Test for Theoretical Maximum Specific Gravity (Gmm) and Density of Asphalt Mixtures.

[93] (2023). Standard Method of Test for Determining Rutting Susceptibility of Asphalt Mixtures Using the Asphalt Pavement Analyzer (APA).

[94] (2024). Standard Test Method for Indirect Tensile (IDT) Strength of Asphalt Mixtures.

