# Supplementary material for: Design and Construction Practices for Full-Depth Reclamation of Asphalt Mixtures with Bituminous and Cementitious Additives
Source: Materials (Basel). 2026 Apr 12;19(8):1540. doi: 10.3390/ma19081540 (PMC13117244; doi:10.3390/ma19081540)
Supplement: Supplementary file 1 [file materials-19-01540-s001.zip › materials-4194070-supplementary.pdf]

APPENDIX A: FDR SURVEY QUESTIONNAIRE

The following sections presents the survey questionnaire to be sent to different highway agencies in the US and worldwide. The topics covered by this survey questionnaire include Full-Depth Reclamation (FDR) specifications, mix design process (with/without bituminous additives), selection of FDR stabilizing agents, construction practices including pavement investigation and selection of the depth of pulverization, field monitoring, performance evaluation, and lessons learned. The main objectives of the survey are: (a) collect information pertaining to FDR from different highway agencies in the US and worldwide, and (b) recommend revisions, if necessary, to the current FDR NJDOT specification. The survey will be distributed using Qualtrics and will take around 10 - 12 minutes to complete.

1.1 Use of Full-Depth Reclamation

1. Please provide your name and title
   - o Name:…………………………….
   - o Title:………………………………
2. Where is your agency located at?
   - 2.1 Country ……………….
   - 2.2 State ……………. (<u>Only if</u> *2.1 Country:* **USA**)

3. What are your main areas of expertise? Please check all that apply.
   - o Constructing rigid/flexible pavements
   - o Pavement preservation
   - o Pavement rehabilitation
   - o Pavement materials
   - o Other (Please specify)

     ………………………………………………………………………………
     ………………………………………………………………………………
     ....

4. Does your agency make use of FDR as a pavement rehabilitation method?
   - o Yes
   - o No
   - o Under Consideration

5. What is the level of knowledge of your agency with Full-Depth Reclamation (FDR) technique?
   - o No knowledge
   - o Little
   - o Good
   - o Excellent

(<u>if</u> *5. What is the level of knowledge of your agency with Full-Depth Reclamation (FDR)*

technique? **No Knowledge,** survey <u>ends</u>)

6. What are the main reasons/benefits your agency uses FDR as a rehabilitation method? Check all that apply.
    - o Cost effectiveness
    - o Environmental benefits
    - o Time saving
    - o Performance improvement
    - o Other? (Please specify)

7. What are the cost savings per lane mile achieved using FDR in your state?
    - o Less than $10,000.
    - o $10,000 – $30,000
    - o $30,000 - $50,000
    - o Over $50,000
    - o No savings

1.2 Mix Design of Full-Depth Reclamation Mixtures

8. How do you collect reclaimed asphalt pavement (RAP) and aggregate base (or soil) when designing FDR mixtures?
    - o Reclaiming/pulverization
    - o Coring
    - o Crushing
    - o Other (Please specify)

      ……………………………………………………………………………………
      ……………………………………………………………………………………
      ……………………………………………………………………………………

9. What is the typical depth of pulverization for your agency when rehabilitating asphalt pavements using FDR?
    - o 5 – 7 in
    - o 7 - 10 in
    - o 10 - 14 in
    - o Other (Please specify)

      ……………………………………………………………………………………
      ……………………………………………………………………………………
      ……………………………………………………………………………………

10. What is the maximum particle size of RAP and aggregate base used for FDR mixtures?
    - o 1/2 inch
    - o 3/4 inch

- 1 inch
- 1.5 inch
- 2 in inch
- Other (Please specify)

  ……………………………………………………………………………………
  ……………………………………………………………………………………
  ……………………………………………………………………………………

11. What are the percentages of RAP and aggregate base allowed in FDR mixtures by your agency?
    - 50% RAP, 50% aggregate base
    - 66.6% RAP, 33.3% aggregate base
    - If there are requirement for selecting the percentages of RAP and aggregate base, please specify:

      ……………………………………………………………………………………
      ……………………………………………………………………………………
      ……………………………………………………………………………………

12. How much corrective aggregates is allowed in the FDR mixtures used by your agency?
    - Less than 5%
    - Between 5% and 15%
    - Between 15% and 25%
    - Over 25%

13. What do you use as chemical additives in FDR mixtures and what is/are their typical percentage(s)?
    - Cement:…………………………
    - Lime slurry:……………………….
    - Liquid anti-strip agent:…………………………….
    - Other additives (Please specify)

      ……………………………………………………………………………………
      ……………………………………………………………………………………
      ……………………………………………………………………………………

14. What is the typical percentage of water used in FDR mixtures, please provide details:
    - 1%
    - 2%
    - 3%
    - Other percentage (please specify):…………………………………..

15. Does your agency allow the use of bituminous additives in FDR mixtures?

- Yes
- No

16. What is/are the bituminous additive/recycling agent used for FDR mixtures as specified by your agency? Check all that apply. (<u>Only if</u> *15. Does your agency allow the use of bituminous additives in FDR mixtures?* **Yes**)
    - Emulsified asphalt
    - Foamed asphalt
    - Asphalt binder
    - Other (Please specify):
      ………………………………………………………………………………………
      ………………………………………………………………………………………
      ………………………………………………………………………………………

17. What are the typical ranges of FDR optimum contents of these stabilizing agents? Please provide additional details for each additive below (if applicable). (<u>Only if</u> *15. Does your agency allow the use of bituminous additives in FDR mixtures?* **Yes**)
    - Emulsified asphalt ……………………………………………………………
    - Foamed asphalt ………………………………………………………………
    - Asphalt Binder ………………………………………………………………
    - Other (Please specify)
      ………………………………………………………………………………………
      ………………………………………………………………………………………
      ………………………………………………………………………………………

18. What compaction method is typically used for compacting FDR mixtures in the laboratory?
    - Gyratory compactor
    - Vibratory compactor
    - Marshall hammer
    - Other? (Please specify)
      ………………………………………………………………………………………
      ………………………………………………………………………………………
      ………………………………………………………………………………………

19. What are the curing period and temperature for FDR mix design purposes in the laboratory?
    - Two days at 60ºC
    - Three days at 60ºC
    - One week at room temperature
    - Other curing process (please specify)
      ………………………………………………………………………………………

…………………………………………………………………………………………
…………………………………………………………………………………………

20. Is the air void level of FDR mixtures determined? If yes, please explain why air void is measured as part of the mix design.
    - o Yes
      …………………………………………………………………………………………
      …………………………………………………………………………………………
      …………………………………………………………………………………………
    - o No

21. What test method is used for measuring the bulk specific gravity of FDR compacted samples? (Only if *20. Is the air void level of FDR mixtures determined? If yes, please explain why air void is measured as part of the mix design?* **Yes**)
    - o Submerging method
    - o Vacuum sealing method

22. What performance tests, if any, you conduct to select the optimum contents of chemical additives and/or binder content for FDR mixtures?
    - o Crack test only
    - o Strength test only
    - o Rut test only
    - o Rut and crack tests
    - o We do not conduct performance testing for FDR mix design.
    - o Other? Please specify the name of the test(s) used:
      …………………………………………………………………………………………
      …………………………………………………………………………………………
      …………………………………………………………………………………………

1.3 Construction Practices of Full-Depth Reclamation

23. What steps are usually taken to select projects for rehabilitation using FDR? In a few sentences, please also provide additional details about what data is collected for this purpose.
    ……………………………………………………………………………………
    ……………………………………………………………………………………
    ……………………………………………………………………………………
    ……………………………………………………………………………………
    ……………………………………………………………………………………

24. What traffic levels for roadways selected for rehabilitation using FDR in your agency?

- Low traffic levels
- Medium traffic levels
- Heavy traffic levels

25. Please specify the range for the selected traffic level:
    - < 3M ESAL
    - 3M - 5M ESAL
    - 5M - 10M ESAL
    - > 10M ESAL
    - Other (please specify)

      ………………………………………………………………………………………
      ………………………………………………………………………………………
      ………………………………………………………………………………………

26. What is the allowable period for curing FDR layers in the field?
    - 1 day
    - 2 – 4 days
    - 5 – 7 days
    - More than a week

27. Please provide additional details about how weather impacts curing of FDR layers in the field (e.g., what are the measures taken when it rains during or one day after the construction of FDR?).

    ………………………………………………………………………………………
    ………………………………………………………………………………………
    ………………………………………………………………………………………
    ………………………………………………………………………………………
    ………………………………………………………………………………………

28. What is the maximum moisture content allowed in FDR layer prior to placing the overlay?
    - Less than 1%
    - 1% – 2%
    - More than 2%
    - No checking for the moisture content

29. How do you measure the moisture content of an FDR layer in the field?

    ………………………………………………………………………………………
    ………………………………………………………………………………………
    ………………………………………………………………………………………
    ………………………………………………………………………………………
    ………………………………………………………………………………………

30. What density do you target when compacting the FDR layer in the field?

- ○ 94% G_{mm}
- ○ 95% G_{mm}
- ○ 96% G_{mm}
- ○ 97% G_{mm}
- ○ Other (please specify)

……………………………………………………………………………………
……………………………………………………………………………………
……………………………………………………………………………………

31. What is the thickness of the overlay placed over the FDR layer in your agency?
    - ○ 1 in. or less
    - ○ 2 – 3 in.
    - ○ 3 – 4 in.
    - ○ At least 4 in.

32. What type of mix/treatment is typically used in your agency to overlay FDR layers?
    - ○ Fine-Graded Dense Hot Mix Asphalt (HMA)
    - ○ Coarse-Graded Dense HMA
    - ○ Open-Graded Friction Courses (OGFC)
    - ○ Stone Matrix Asphalt (SMA)
    - ○ No top layer is needed.
    - ○ Other (please specify)

……………………………………………………………………………………
……………………………………………………………………………………
……………………………………………………………………………………

33. Does your agency require the contractor to conduct quality control?
    - ○ Yes
    - ○ No

34. What are the quality control requirements the contractor is mandated to perform on FDR? Please check that all applies. (Only if *33. Does your agency require the contractor to conduct quality control??* **Yes**)
    - ○ In-Place Density
    - ○ Layer thickness
    - ○ Gradation
    - ○ Binder content
    - ○ Other (please specify)

……………………………………………………………………………………
……………………………………………………………………………………
……………………………………………………………………………………

35. What are the measures followed by your agency to ensure the quality of FDR (quality acceptance)? Please check that all applies.
   - o In-Place Density
   - o Layer thickness
   - o Gradation
   - o Binder content
   - o Other (please specify)

   ……………………………………………………………………………………
   ……………………………………………………………………………………
   ……………………………………………………………………………………

36. Based on your experience, how do you rate the performance of pavements rehabilitated using FDR **<u>one</u>** year after construction?
   - o Excellent Performance (No distresses)
   - o Good Performance (distresses are minor)
   - o Poor Performance (distresses are abundant)

37. Based on your experience, how do you rate the performance of pavements rehabilitated using FDR **<u>five</u>** years after construction?
   - o Excellent Performance (No distresses)
   - o Good Performance (distresses are minor)
   - o Poor Performance (distresses are abundant)
   - o Not applicable (No data are available)

38. Based on your experience, how do you rate the performance of pavements rehabilitated using FDR **<u>10</u>** years after construction?
   - o Excellent Performance (No distresses)
   - o Good Performance (distresses are minor)
   - o Poor Performance (distresses are abundant)
   - o Not applicable (No data available)

39. List the challenges, if any, encountered during the construction process of FDR (e.g., project selection, milling, paving, compaction, etc…).

   ……………………………………………………………………………………
   ……………………………………………………………………………………
   ……………………………………………………………………………………
   ……………………………………………………………………………………
   ……………………………………………………………………………………

40. How do you overcome these challenges?

   ……………………………………………………………………………………
   ……………………………………………………………………………………
   ……………………………………………………………………………………

…………………………………………………………………………………
…………………………………………………………………………………

41. In one or few sentences, what are the main lessons learned from the FDR projects conducted in your agency?

…………………………………………………………………………………
………………………………………………………………………………...
…………………………………………………………………………………
…………………………………………………………………………………
…………………………………………………………………………………
